# Quantification of the three-dimensional root system architecture using an automated rotating imaging system

**DOI:** 10.1186/s13007-023-00988-1

**Published:** 2023-02-02

**Authors:** Qian Wu, Jie Wu, Pengcheng Hu, Weixin Zhang, Yuntao Ma, Kun Yu, Yan Guo, Jing Cao, Huayong Li, Baiming Li, Yuyang Yao, Hongxin Cao, Wenyu Zhang

**Affiliations:** 1 IGRB-IAI Joint Laboratory of Germplasm Resources Innovation & Information Utilization, YuanQi-IAI Joint Laboratory for Agricultural Digital Twin, Institute of Agricultural Information, Jiangsu Academy of Agricultural Sciences, Nanjing, 210014 Jiangsu China; 2 Plant Phenomics Research Center, Academy for Advanced Interdisciplinary Studies, Nanjing Agricultural University, Nanjing, 210095 Jiangsu China; 3 School of Agriculture and Food Sciences, The University of Queensland, St. Lucia, QLD 4072 Australia; 4 School of Agricultural Engineering, Jiangsu University, Zhenjiang, 212013 Jiangsu China; 5 College of Land Science and Technology, China Agricultural University, Beijing, 100193 China; 6 IGRB-IAI Joint Laboratory of Germplasm Resources Innovation & Information Utilization, Institute of Germplasm Resources and Biotechnology, Jiangsu Academy of Agricultural Sciences, Nanjing, 210014 Jiangsu China; 7 College of Electronics & Information Engineering, Nanjing University of Information Science and Technology, Nanjing, 210044 Jiangsu China

**Keywords:** Automated imaging, Multi-view stereo, 3D root phenotyping, Global/local root trait, Root segmentation, Initial root angle

## Abstract

**Background:**

Crop breeding based on root system architecture (RSA) optimization is an essential factor for improving crop production in developing countries. Identification, evaluation, and selection of root traits of soil-grown crops require innovations that enable high-throughput and accurate quantification of three-dimensional (3D) RSA of crops over developmental time.

**Results:**

We proposed an automated imaging system and 3D imaging data processing pipeline to quantify the 3D RSA of soil-grown individual plants across seedlings to the mature stage. A multi-view automated imaging system composed of a rotary table and an imaging arm with 12 cameras mounted with a combination of fan-shaped and vertical distribution was developed to obtain 3D image data of roots grown on a customized root support mesh. A 3D imaging data processing pipeline was developed to quantify the 3D RSA based on the point cloud generated from multi-view images. The global architecture of root systems can be quantified automatically. Detailed analysis of the reconstructed 3D root model also allowed us to investigate the Spatio-temporal distribution of roots. A method combining horizontal slicing and iterative erosion and dilation was developed to automatically segment different root types, and identify local root traits (e.g., length, diameter of the main root, and length, diameter, initial angle, and the number of nodal roots or lateral roots). One maize (*Zea mays* L.) cultivar and two rapeseed (*Brassica napus* L.) cultivars at different growth stages were selected to test the performance of the automated imaging system and 3D imaging data processing pipeline.

**Conclusions:**

The results demonstrated the capabilities of the proposed imaging and analytical system for high-throughput phenotyping of root traits for both monocotyledons and dicotyledons across growth stages. The proposed system offers a potential tool to further explore the 3D RSA for improving root traits and agronomic qualities of crops.

**Supplementary Information:**

The online version contains supplementary material available at 10.1186/s13007-023-00988-1.

## Background

The root system is responsible for the absorption of soil water and nutrients and the anchorage of plants [1]. Ideal root architecture maximizes the uptake efficiency of soil nutrients and thus increases crop photosynthesis and yield. Three-dimensional (3D) root system architecture (RSA) plays an essential role in genotype selection for crop breeding [2]. Identifying, evaluating, and selectively introducing dominant root traits into breeding programs may be a promising area for improving food security in developing countries [3]. However, due to the opacity of the root growth medium and RSA complexity, the observation and identification of relevant quantitative phenotypes of root systems are difficult.

Traditional root measurement methods are time-consuming and destructive, so interest in high-throughput root phenotyping platforms is increasing in public and private research. High-throughput automated root phenotyping platforms have been developed to obtain two-dimensional (2D) images of roots grown in soil/non-soil media [4–6]. Due to cost-effective sensors, a high degree of automation, easy scaling of throughput, and convenient operation, these platforms are widely used for the quantification of high-throughput root phenotyping and the selection of dominant root traits. However, the limited growing space may have a great effect on RSA, and many root traits are difficult to quantify in 2D.

3D representations of root systems in controlled environments can now be produced using non-destructive 3D root phenotyping systems. Optical imaging systems can quantify the 3D growth dynamics of young roots grown in transparent media [7, 8]. X-ray computed tomography (X-ray CT) [9, 10], magnetic resonance imaging (MRI) [11], and neutron radiography [12] enable in situ observation of the 3D RSA growing naturally in soil medium. Root models derived from MRI and X-ray CT images enable the quantification of root growth over time and the acquisition of spatiotemporal 4D RSA in the laboratory [13–15]. However, the application of these methods is limited by shortcomings such as high cost, technical difficulties, time-consuming imaging process, throughput expansion difficulties, and the limited size of cultivation containers.

Methods for root phenotyping under controlled conditions that are mainly applicable to plant seedlings may not reliably reproduce results expected in field conditions, particularly in the reproductive stage [16, 17]. Field-based phenotyping at the late growth stage can provide insights into post-embryonic root structures of mature field-grown plants [16]. At present, a technical bottleneck still exists in the observation of mature roots of field-grown plants. Invasive root tracing involves the transparent surface of a buried minirhizotron with a cylindered imaging sensor, but only a small proportion of roots is visible [18]. Shovelomics, a destructive method for the quantification of mature field-grown roots, can be used to acquire measurements by excavation, 2D imaging, and automated image processing [19, 20]. Recently, DIRT/3D, an image-based root phenotyping platform, successfully measured root traits from mature field-grown maize (*Zea mays* L.) root crowns extracted by the shovelomics method [21]. Although the sampling was destructive, the extraction efficiency of root traits was improved with high-throughput measurements using an automated imaging system and root analysis software. However, data on roots obtained by this method was incomplete (e.g., only root crowns close to the root base were quantified). To span, the gap between the laboratory and field, the root mesocosm system, which allows for unconstrained root growth, excavation, and preservation of completeness 3D RSA, was developed [22]. However, this approach comes at the cost of labor and space.

Currently, available methods are all imperfect as each has its own key objective and spectrum of trade-offs. 2D root phenotyping and 3D gel optical platforms achieve low-cost, high-throughput root phenotyping at the expense of the natural growth environment. X-ray CT and MRI enable in-situ observation of the 3D RSA growing naturally in soil medium at the expense of equipment cost and throughput. Shovelomics and DIRT3D enable the rapid acquisition of root phenotypes in the field at the expense of root integrity. To provide a balance among throughput, natural growth medium, cost, the integrity of RSA, and observable period, we provided a root growth system and developed an automated multi-view imaging system and data processing pipeline for the quantification of the 3D RSA at different stages for both monocotyledonous and dicotyledonous crops grown in field-like growth medium. One maize (*Zea mays* L.) and two rapeseed (*Brassica napus* L.) cultivars at different growth stages were selected to evaluate the performance of our system. The root growth system struck a balance among field-like growth medium, preservation of 3D RSA, relatively low constraints on root growth, ease of handling, and low costs. The automated imaging system improved the capture of 3D root phenotypic information while also reducing the cost of labor and investment. The global and local root traits can be automatically extracted by the proposed pipeline, which can balance the simplicity of global traits of the whole root system and the elaboration of local traits of different root types for comprehensive 3D RSA analysis.

## Results

### Development of an automated imaging and 3D reconstruction pipeline

**Fig. 1:**
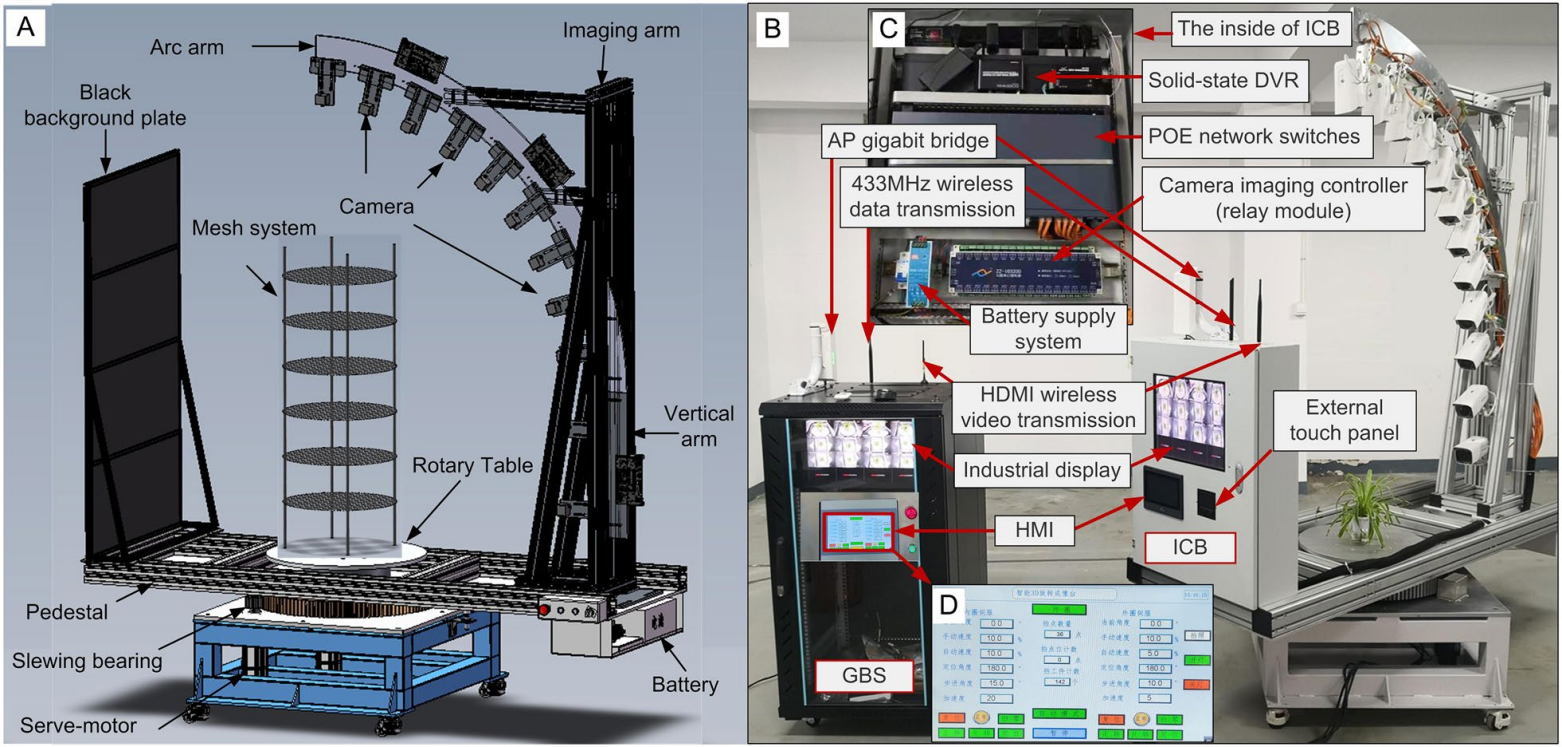
Automated imaging system. **A** 3D representation of the components of the automated imaging system, including a rotary table, an imaging arm, a pedestal, and a black background plate. **B** Automated imaging device prototype. An imaging control box (ICB) was mounted on the opposite side of the imaging arm, and a ground base station (GBS) was configured. **C** ICB components. **D** Human–machine interface (HMI) on the GBS

**Fig. 2.**
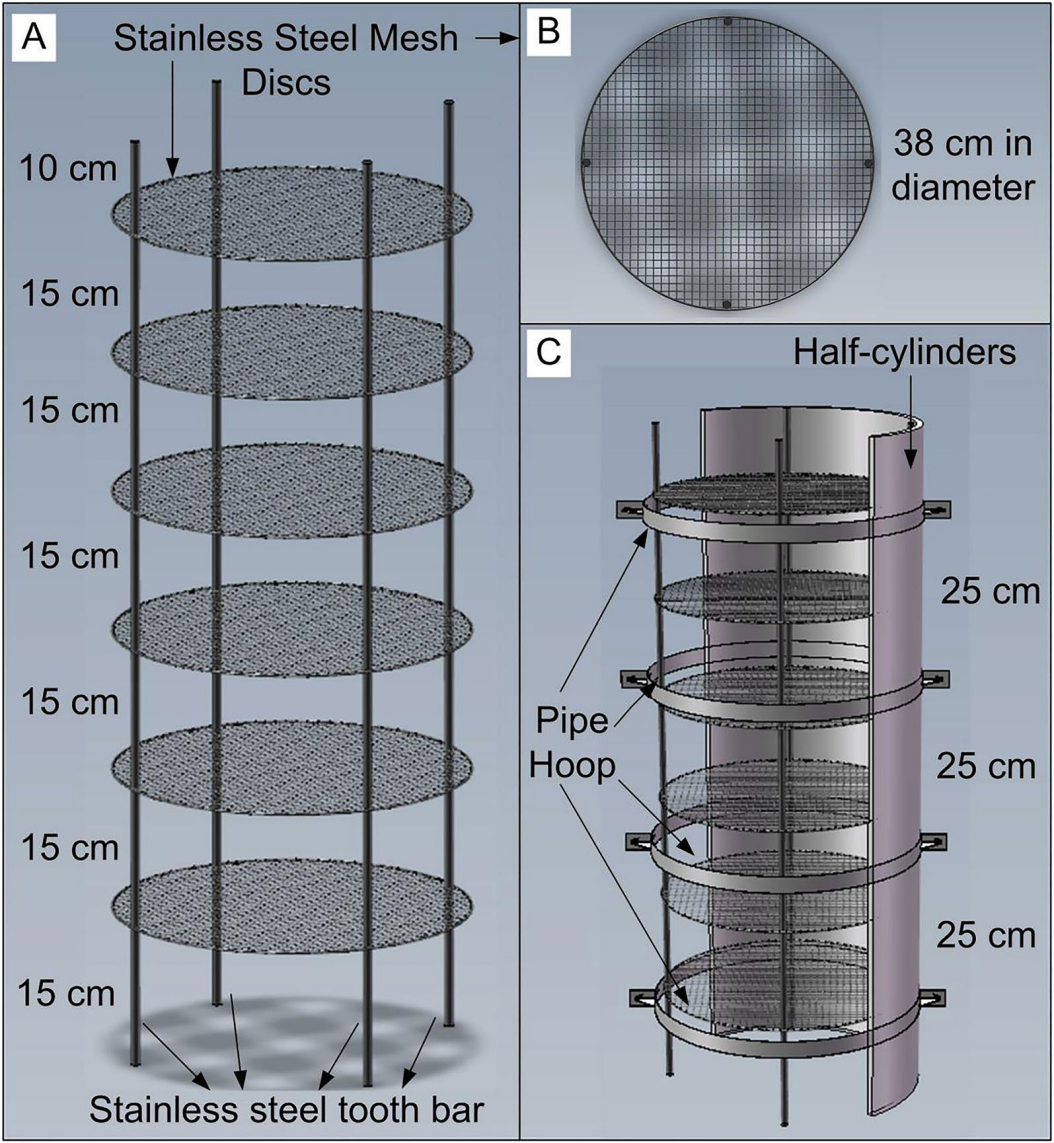
3D representation of the components of the custom-made root growth system. **A** Custom-made root support mesh. **B** Top view of a stainless steel mesh disc. **C** Root support mesh, half-cylinders, and four pipe hoops

**Fig. 3:**
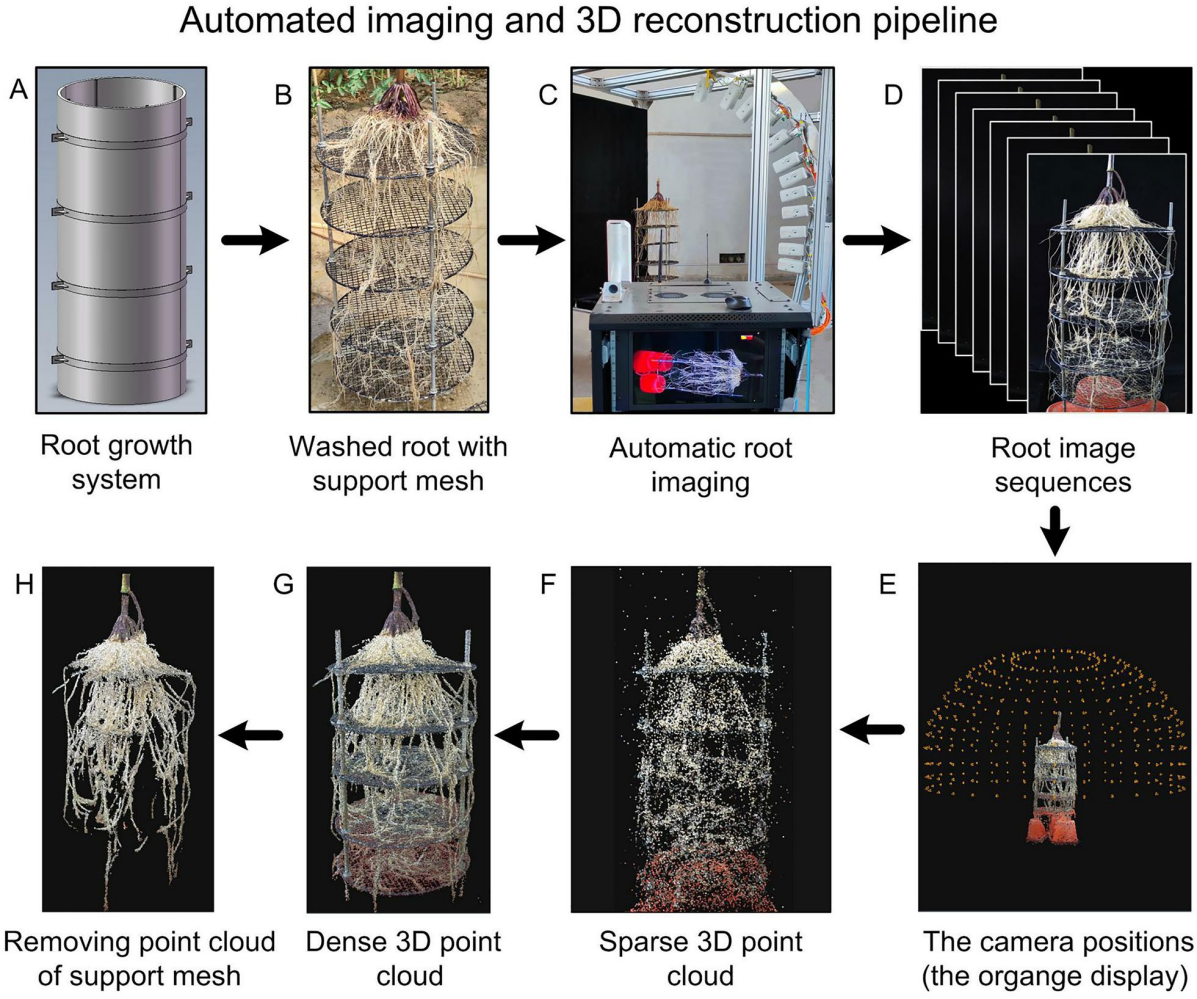
Automated imaging and 3D reconstruction pipeline. **A** Root growth system. **B** Washed root held by the support mesh system. **C** Automated imaging system. **D** Automatically obtained multi-view images. **E** Hemispherical distribution of camera imaging positions. **F** Generated sparse 3D point cloud. **G** Generated dense 3D point cloud. **H** 3D point cloud without the support mesh

A multi-view automated imaging system composed of a rotary table and an imaging arm with 12 cameras mounted with a combination of fan-shaped and vertical distribution was developed to obtain 3D image data of the root system (Fig. 1). A root growth system was constructed to cultivate plants and retain the root structure (Figs. 2 and 3A, B). All 432 images with hemispherical distribution around the root system were obtained within 3 min (cameras performed imaging with each 10° rotation of the imaging arm) using the automated imaging system (Fig. 3C, D). The structure-from-motion and multi-view stereo (SFM-MVS) pipeline was used to generate dense 3D point clouds of root systems from multi-view images. SFM-MVS is related to using mathematical techniques to recover the 3D scenes and objects viewed from multiple positions (multi-view) of cameras. The SFM technique was first used to conduct an alignment of the multi-view images for calculating the epipolar geometry of the scenes and thus generating the sparse 3D point cloud (e.g., feature points; Fig. 3F), the camera positions (Fig. 3E) as well as the internal parameters of the camera lenses (e.g., focal length, radial, and tangential distortion coefficients). The MVS algorithm [23, 24] was then employed to generate the dense point clouds (Fig. 3G) given the information related to epipolar geometry calculated by the SFM technique. The MVS algorithm operated on all the pixel values, which enabled the recovery of the majority of geometric details in the scenes. The 3D point cloud of the black root support mesh was removed by chromatic aberration denoising (Fig. 3H).

**Fig. 4:**
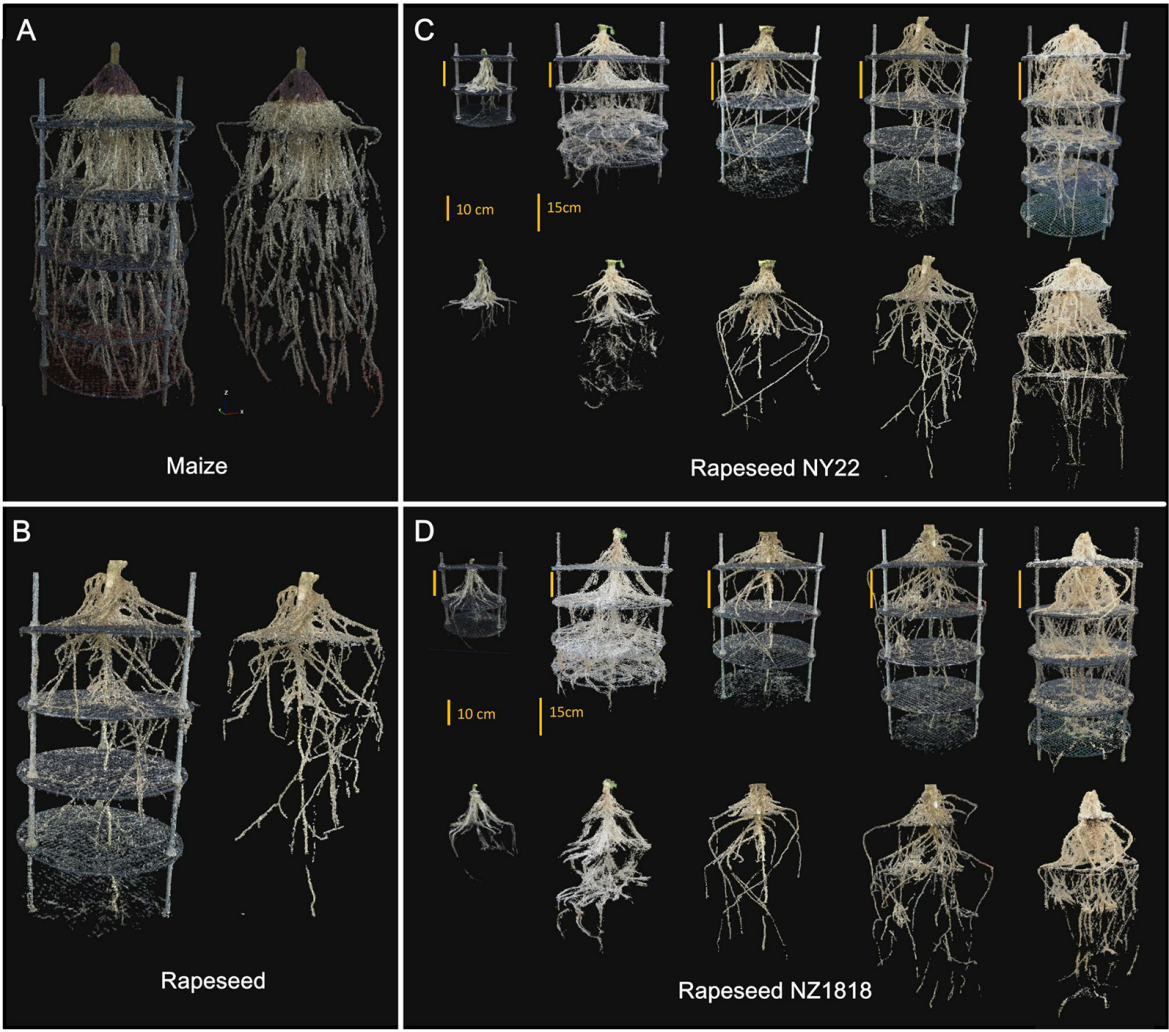
Reconstructed 3D point clouds of root systems with and without the root support mesh for mature maize (**A**), mature rapeseed (**B**), and two rapeseed cultivars (NY22 and NZ1818) at different growth stages (**C**, **D**)

The automated imaging system and 3D reconstruction pipeline enabled the high-efficiency acquisition of multi-view images and high-precision reconstruction of 3D point clouds of root systems (Fig. 3). The visual comparison of the captured 3D RSA (with and without the root support mesh) of two crops (maize and rapeseed) and the variation between two rapeseed cultivars (NY22 and NZ1818) at different growth stages is shown in Fig. 4.

### Quantitative analysis of the global root architecture

**Fig. 5:**
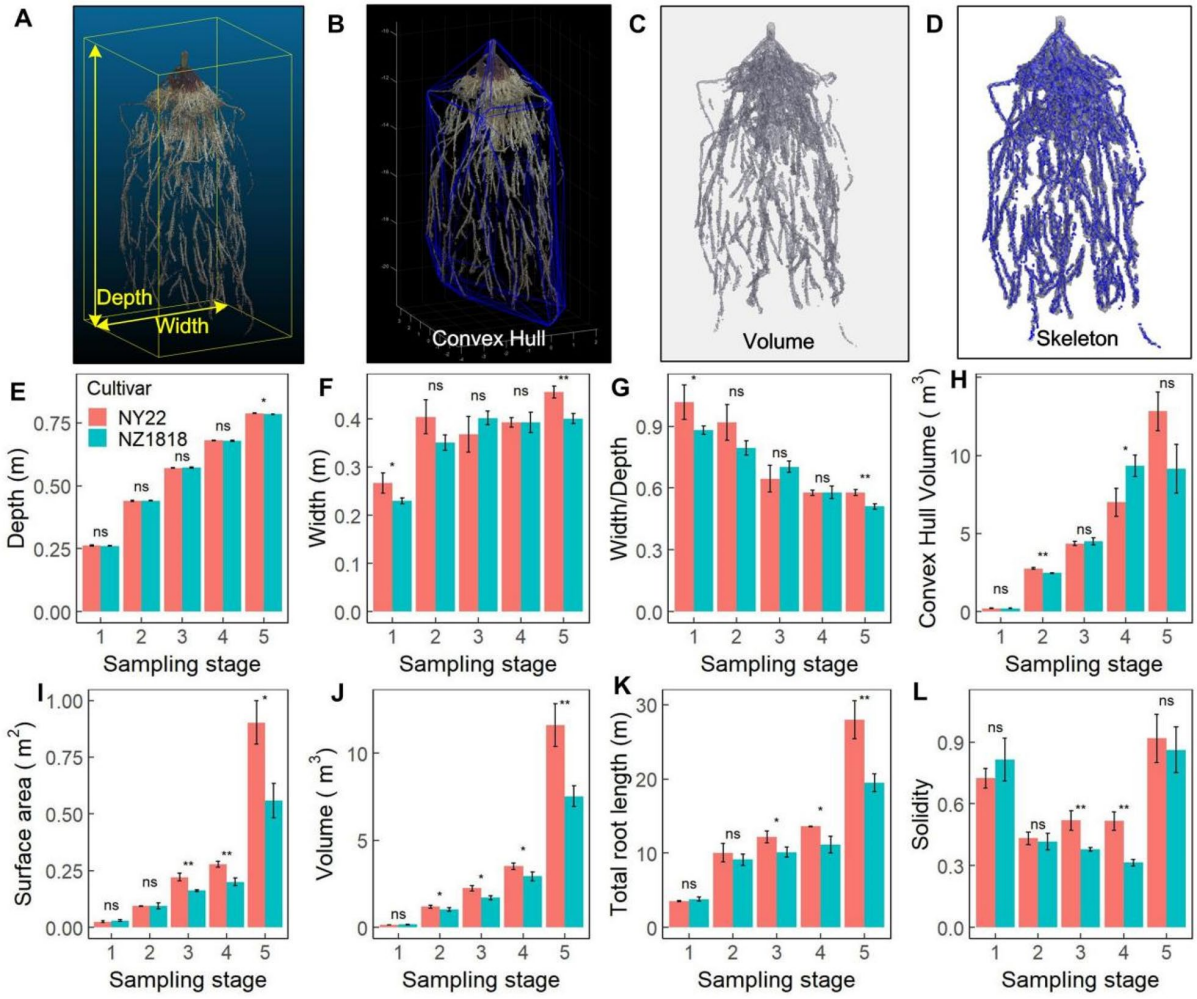
Quantification of global root traits. Extraction pipeline of global root traits of root depth and width (**A**), convex hull volume (**B**), area and volume (**C**), and skeleton and total length (**D**) of mature maize. Root depth (**E**), width (**F**), width/depth (**G**), convex hull volume (**H**), surface area (**I**), volume (**J**), total length (**K**), and solidity (**L**) for two rapeseed cultivars (NY22 and NZ1818) at different stages. Significant differences were assessed from three repeats by standard *t*-tests (*p < 0.05, **p < 0.01, ***p < 0.001)

The reconstructed 3D point cloud of the root system allowed us to assess a variety of root traits. A customized 3D point cloud processing pipeline was developed to automatically extract the global root traits of root depth, width, width/depth, convex hull volume (CHV), volume (V), surface area (SA), solidity (V/CHV), and total root length (TRL) (Fig. 5A–D). To examine the 3D RSA of rapeseed, we extracted the global root traits of two rapeseed cultivars at five growth stages (Fig. 5E–L). The root width exhibited a characteristic S-shaped curve because of the limitations of the containers (Fig. 5F). The root depth, convex hull volume, surface area, volume, and total root length (Fig. 5E, H–K) increased with growth stages, whereas the width/depth ratio (Fig. 5G) decreased with growth stages. The solidity was higher at the 1st and 5th sampling stages and lower at the 2nd–4th sampling stages (Fig. 5L). The root surface area, volume, and total length were higher in NY22 than in NZ1818 at the 4th–5th sampling stages, indicating that NY22 may have denser root growth than NZ1818 (Fig. 5I–L). The automated extraction pipeline of global root traits was suitable for both monocotyledonous (maize) and dicotyledonous (rapeseed) roots (Fig. 5 and Additional file 1: Fig. S1).

**Fig. 6:**
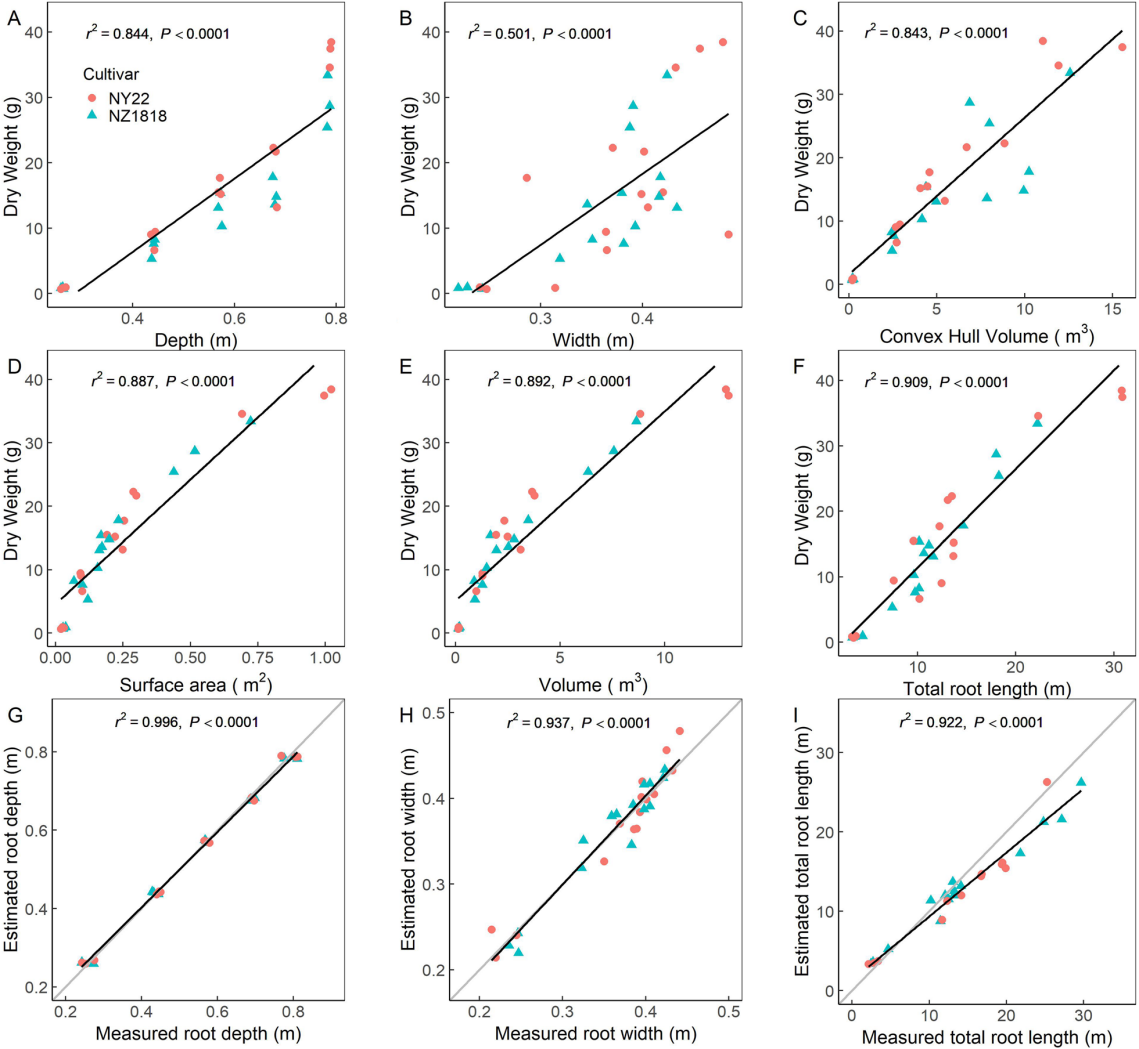
Correlations of extracted global root traits and root dry weight (**A**–**F**) and correlations of extracted and measured global root traits (**G**–**I**). Root depth (**A**), width (**B**), convex hull volume (**C**), surface area (**D**), volume (**E**), and total root length (**F**) correlated with manually weighed dry weight; and the estimated root depth (**G**), width (**H**), and total root length (**I**) correlated with manually measured values. Red and blue samples indicated NY22 and NZ1818 rapeseed cultivars, respectively

The relationships between the extracted global root traits and the dry weight of root systems, and between the extracted and measured global root traits were investigated. All root depth, convex hull volume, surface area, volume, and total length were significantly correlated with dry weight (*r*^*2*^ > 0.8, *P* < 0.0001; Fig. 6A, C–F). The *r*^2^ values of root surface area, volume, and total length were higher than those of convex hull volume and root depth. A strong linear relationship was observed between the measured and extracted root depth and root width (*r*^*2*^ > 0.9, *P* < 0.0001; Fig. 6G, H), so did the relationship between the measured and extracted total root length (*r*^*2*^ > 0.9, *P* < 0.0001; Fig. 6I). However, there were a few outliers, which suggested that certain mismatches existed between the extracted and measured values, and the relative error was slightly larger for root systems at the late growth stage.

### The spatio-temporal distribution of root system

**Fig. 7:**
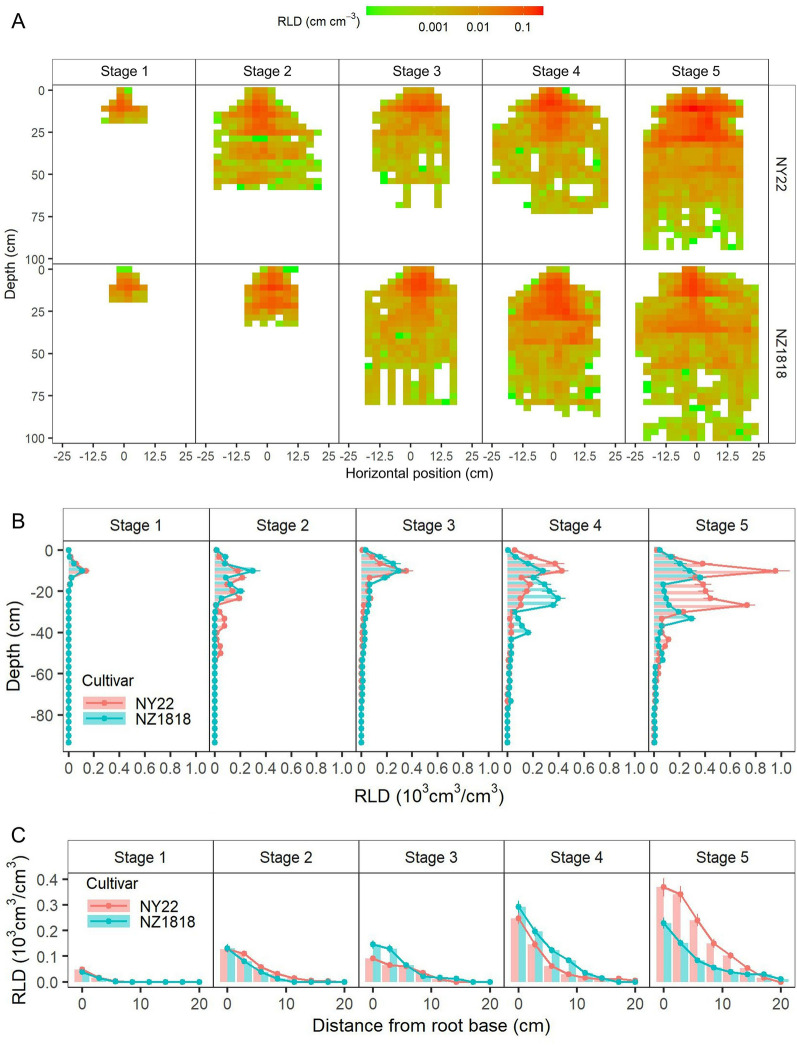
The Spatio-temporal distribution of root length density (RLD). The 3D spatial distribution of RLD (**A**), and the vertical (**B**) and horizontal (**C**) distribution of RLD for two rapeseed cultivars (NY22 and NZ1818) at different growth stages. The position of the stem base was the original 0

Based on the reconstructed 3D root model, the spatial distribution of root density was analyzed by calculating the root length in non-overlapping cubes of approximately 3 cm^3^ encompassing the whole root system. Root length density (RLD) was analyzed separately for mature maize and rapeseed (Additional file 1: Fig. S2). Further, the 3D spatial distribution of root systems of two rapeseed cultivars from the vegetative to the reproductive stage was analyzed (Fig. 7A). Root density was higher in the region of 30 cm deep and 10 cm wide from the root base. From the seedlings stage (stage 1) to the flowering stage (stage 5), the soil space occupied by roots gradually increased. NY22 occupied soil space faster in the early stage (stage 2). While, NZ1818 occupied more soil space in the late stage (stage 5), and had a relatively sparse root distribution.

The vertical and horizontal RLD distribution of individual plant roots were quantified for two rapeseed cultivars from the vegetative to the reproductive stage (Fig. 7B and C). With increasing soil depth, RLD first increased and then decreased (Fig. 7B). The RLD all peaked at a depth of 10 cm, except for NZ1818 at stage 4. No significant differences were found between the RLD profiles of the two cultivars at stages 1–3. Compared with NZ1818, the RLD of NY22 was greater in the top 10 cm but lower at depths below 20 cm at stage 4, and was greater in the top 30 cm at stage 5. The RLD decreased with increasing horizontal distance from the root base for both cultivars (Fig. 7C). No significant differences were found between the RLD horizontal distributions of the two cultivars at stages 1–2. Compared with NZ1818, the RLD of NY22 was lower at 0–5 cm away from the root base at stage 3 and 0–10 cm away from the root base at stage 4 but was greater at 0–15 cm away from the root base at stage 5.

### Combination of horizontal slicing and erosion dilation enables the segmentation of different root types

**Fig. 8:**
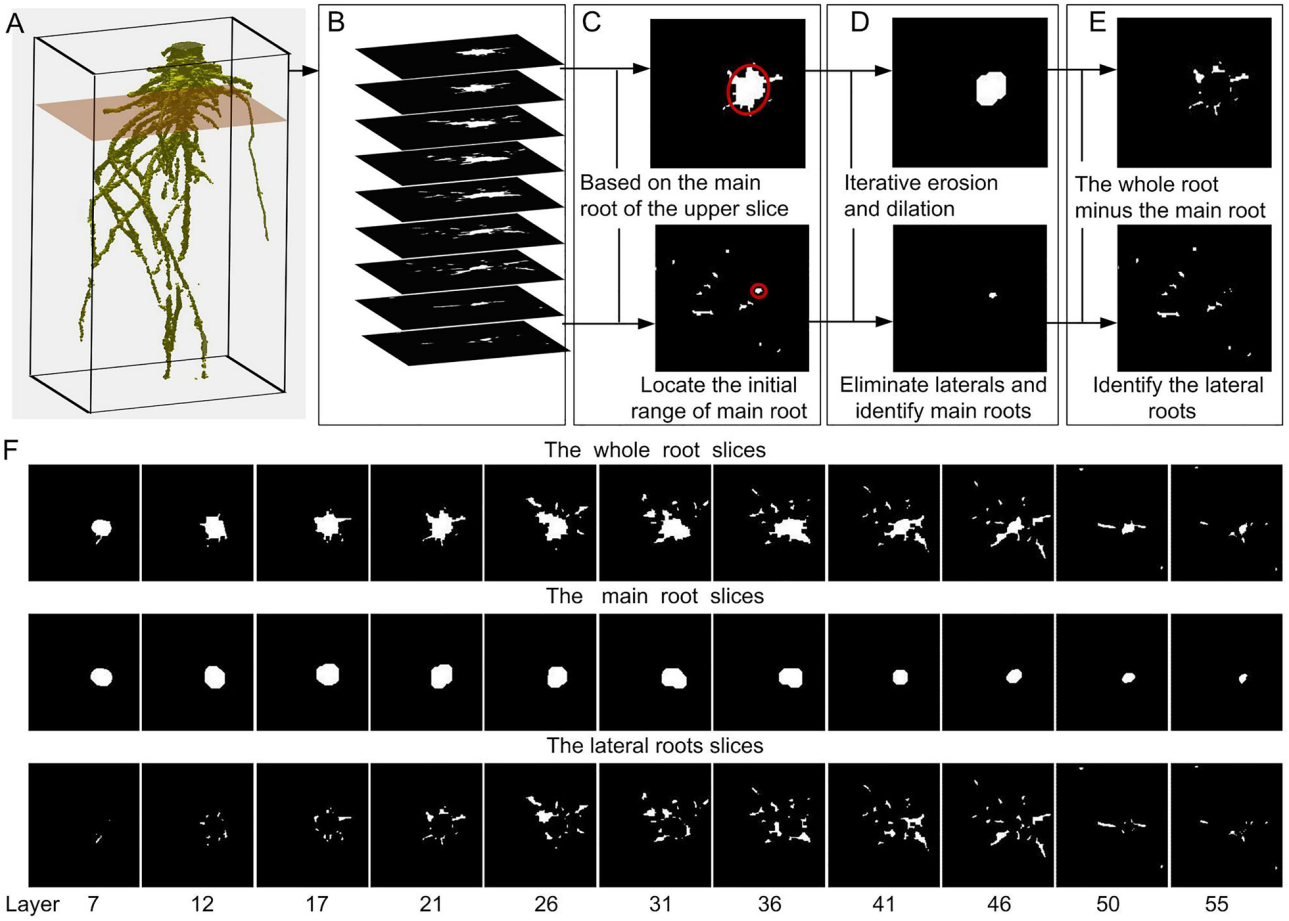
Automated root segmentation pipeline. **A** A sliding plane slices the 3D root model from top to bottom at consecutive depth levels. **B** A level set image sequence of 2D projection slice of roots. **C** The initial range of main root location. **D** Elimination of lateral roots and identification of the main root. **E** Identification of lateral roots. **F** A slice sequence of the automatically segmented main root and lateral roots

A method combining horizontal slicing and iterative erosion and dilation was developed to automatically segment different types of roots (e.g., segment the lateral roots from the main root of rapeseed, or segment the nodal roots from the stem of maize). Take rapeseed, for example, the 3D root model was sliced from top to bottom at consecutive depth levels (Fig. 8A), and a level-set image of a 2D projection slice of roots was obtained (Fig. 8B). The initial region for the main root identification on each slice was determined according to the main root on the upper slice, which is the range of the main root on the upper slice dilated by 10% of its area (Fig. 8C). An iterative erosion and dilation algorithms (imerode and imdilate [25]) were used to eliminate lateral root branches and identify the main root (Fig. 8D). The number of iterations and magnitude of erosion and dilation was determined by the area of roots on each slice. The lateral roots were obtained using the whole root minus the main root (Fig. 8E). The results show that the developed method was effective in the automatic segmentation of different root types (Fig. 8F). To obtain a complete and continuous main root, each slice was sequentially detected to locate the fractured blank slice layers and connect the voxels of two non-blank slice layers.

### Root segmentation enables automated extraction of local root traits

**Fig. 9:**
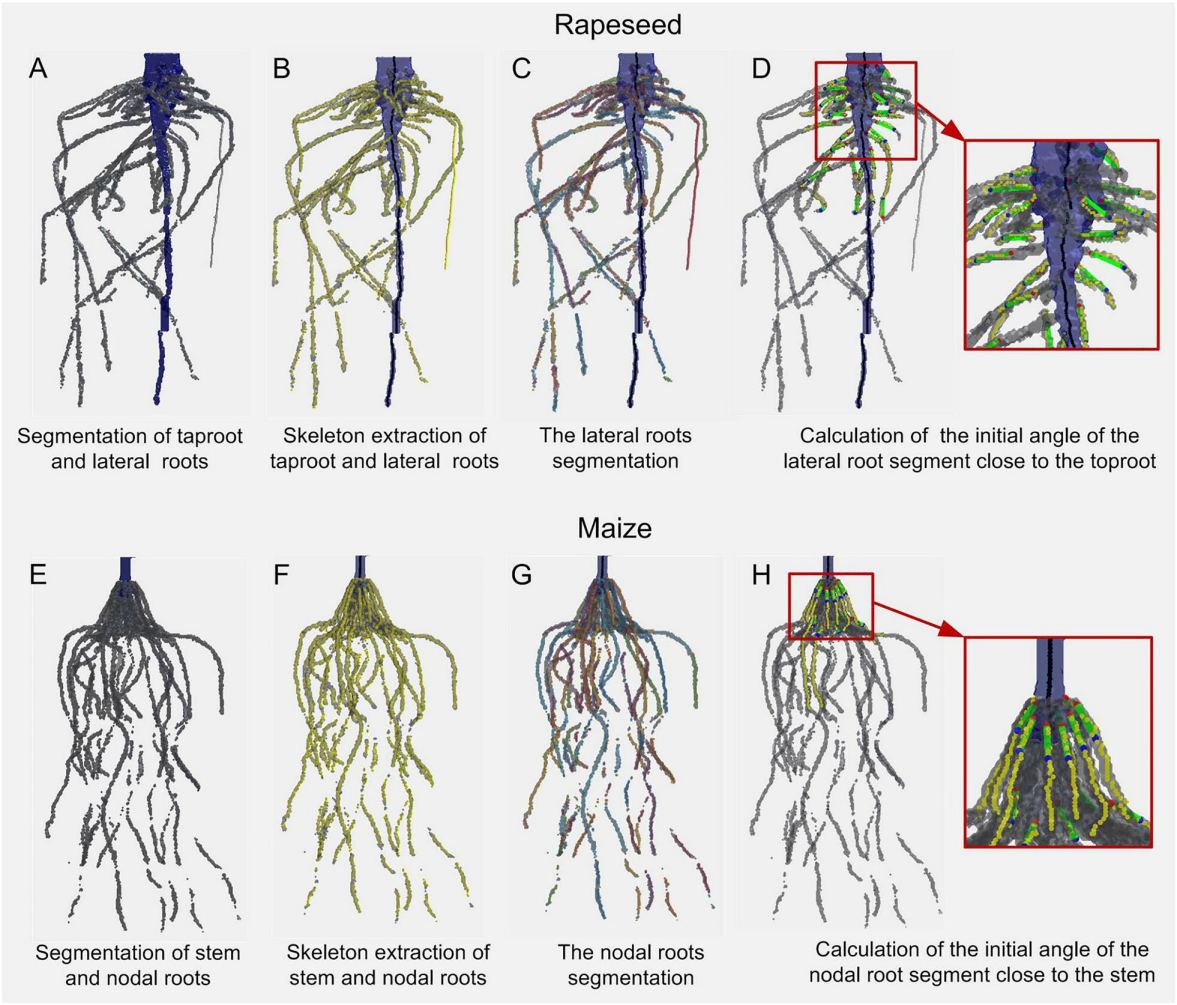
Local root traits extraction pipeline for rapeseed (**A**–**D**) and maize (**E**, **F**), respectively. **A**, **E** Segmentation of different root types. The blue voxel indicated the main root/stem; the dark gray voxel indicated the lateral/nodal roots. **B**, **F** Skeleton extraction of different root types. The black line indicated the main root/stem skeleton; the yellow line indicated the skeleton of lateral/nodal roots. **C**, **G** Root segmentation. Divided lateral/nodal root segments are indicated in different colors. **D**, **H** Measurement of the initial angle. Root segments selected for initial angle measurement are indicated with yellow lines, start points are indicated in red, point 10 voxels away from the start point is indicated in blue, and the vector of the initial angle is indicated by the green line

Local root traits were extracted automatically using algorithms developed in the 3D imaging data processing pipeline. These traits were length, the average diameter of the main root or lateral roots, and initial angle of lateral roots for dicotyledons (rapeseed) (Fig. 9A–D), and the total length, average diameter, initial angle, and the number of nodal roots and stem diameter for monocotyledons (maize) (Fig. 9E–F). The lateral roots were segmented from the main root of rapeseed, and the nodal roots were segmented from the stem of maize, using the developed root segmentation method (Figs. 8 and 9A, E). The skeleton of the roots was extracted using the optimized homotopic thinning algorithm (Fig. 8B, F). The branching points of the 3D skeleton were extracted using the “branchpoints” operation in *bwmorph3*, and the 3D skeleton of lateral roots was divided into numerous root segments by removing the branching points (Fig. 9C, G). To calculate the initial angle and the number of lateral roots or nodal roots, the root segments emerging from the main root or stem needed to be selected first (Fig. 9D, H). The closest point to the main root or stem of each root segment was selected and marked as the start point (red points in Fig. 9), and then whether the distance of the point to the skeleton of the main root or stem was less than the maximum diameter of the main root or stem was determined. Eligible root segments were selected to calculate the number and initial angle of root segments. The initial angle of a root segment was the vertical angle of the vector (green line in Fig. 9) from the point 10–15 voxels away from the start point (blue points in Fig. 9).

**Fig. 10:**
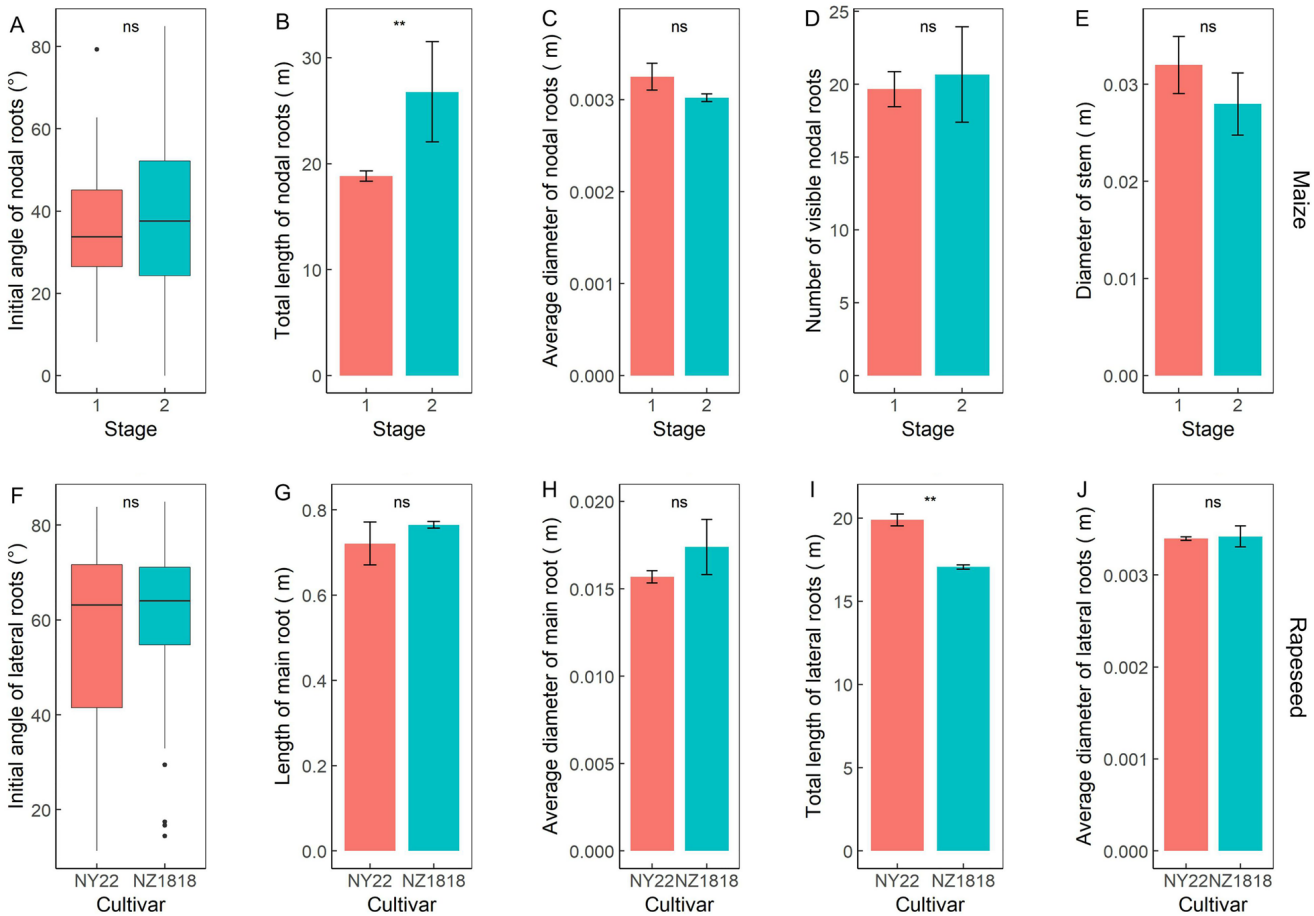
Quantification of local root traits. Initial angle (**A**), total length (**B**), the average diameter (**C**), visible number of nodal roots (**D**), and stem diameter (**E**) for mature maize at two sampling stages (filling stage and mature stage). Initial angle (**F**), total length (**I**), and average diameter (**J**) of lateral roots, and length (**G**) and average diameter (**H**) of the main root for two cultivars (NY22 and NZ1818) of mature rapeseed. Significant differences were assessed from three repeats by standard t-tests (*p < 0.05, **p < 0.01, ***p < 0.001)

The local root traits were extracted for mature maize at two growth stages and for two rapeseed cultivars (Fig. 10). For maize roots, no significant differences were found between the filling stage and mature stage in the initial angle, average diameter, the number of visible nodal roots, and stem diameter (Fig. 10A, C–E), whereas the total length of nodal roots increased significantly (*P* < 0.001; Fig. 10B). For the two rapeseed cultivars, no significant differences were observed between NY22 and NZ1818 in the initial angle, the average diameter of lateral roots, and the length and average diameter of the main root (Fig. 10F–H, J), whereas the total length of lateral roots of NY22 was significantly higher than that of NZ1818 (*P* < 0.001; Fig. 10I). Variations in the initial angle of lateral roots were higher in NY22 (*SD* = 21.74, *C*·*V* = 38.80%) than in NZ1818 (*SD* = 15.92, *C*·*V* = 25.98%; Fig. 10F). In addition, the initial angle was steeper in nodal roots of maize than in lateral roots of rapeseed (Fig. 10A, F). As measured from the vertical direction, the former was in the range 30–50° (*SD* = 13.83, *C*·*V* = 38.11%), whereas the latter was concentrated at 45°–70° (*SD* = 18.42, *C*·*V* = 47.32%).

**Fig. 11:**
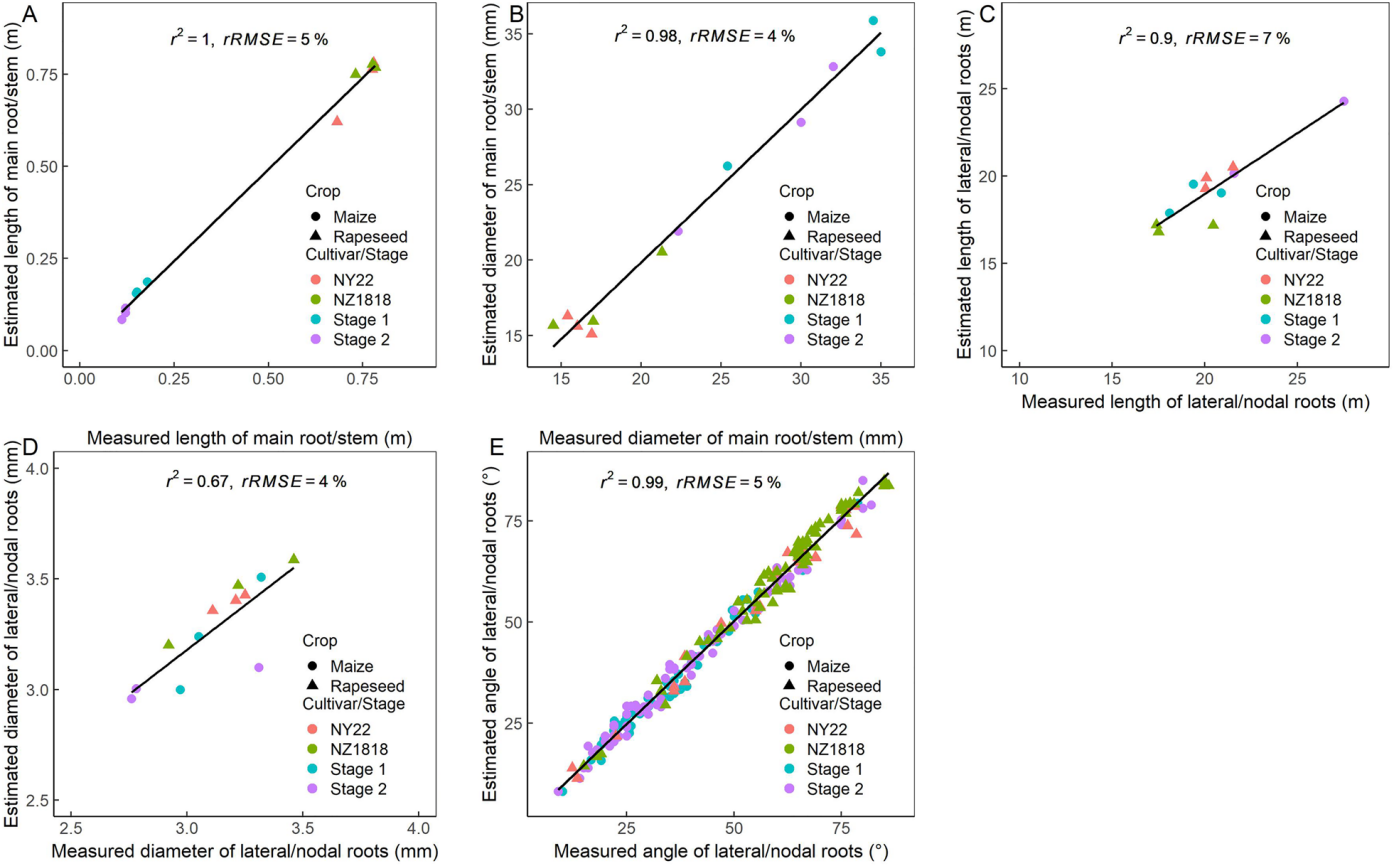
Comparisons of the length (**A**) and diameter (**B**) of the main root of rapeseed and stem of maize, and the length (**C**), diameter (**D**), and initial angle (**E**) for the lateral roots of rapeseed and nodal roots of maize between manual measurement and estimation from the reconstructed root systems

The local root traits that were extracted from the 3D point clouds showed linear relationships with their manually measured values for the length and diameter of the main root of rapeseed and stem of maize (*r*^*2*^ > 0.98, *P* < 0.0001, rRMSE < 5%; Fig. 11A and B), and the length, diameter and initial angle for the lateral roots of rapeseed and nodal roots of maize (*r*^*2*^ > 0.67, *P* < 0.0001, rRMSE < 7%; Fig. 11C–E).

## Discussion

### Automated imaging system and reconstruction pipeline facilitates 3D root phenotyping

The root growth system consisting of PVC pipes and supporting mesh adopted in this study provided a relatively large space for root growth and facilitated the maintenance of the 3D architecture of root systems. While it could be inferred from the reconstructed 3D point cloud of rapeseed (Fig. 4) that the root system of rapeseed was limited by the size of the container. A larger container was required to obtain a more precise growth trajectory for the lateral roots of rapeseed.

The multi-view automated imaging system proposed in this study could efficiently obtain 3D RSA information for maize and rapeseed. Such information can be of great value for advances in high-throughput 3D root imaging. Although 2D high-throughput root imaging systems are more accessible to the wider scientific community, a multi-dimensional understanding of root biology and mapping genes controlling complex topologies may still require the precision architecture afforded by 3D imaging [8].

Among plant 3D reconstruction technologies, digital photography techniques provide an economic, efficient, and convenient way to generate a 3D point cloud for plant phenotyping. However, the manual acquisition of multi-view images is time-consuming and labor-intensive as hundreds of images are usually required to generate high-quality 3D point clouds. The proposed automated imaging system minimized intervention and provided efficient acquisition of multi-view images (432 images shot within 3 min per plant), significantly reducing the cost of labor and equipment. In addition, by precisely controlling the vertical and azimuth angles of imaging views, the automated imaging system could largely avoid the failure of 3D reconstruction, which often occurred when using manually acquired and unevenly distributed images (Additional file 1: Fig. S3).

Current installations of SFM scanners with multiple cameras were designed based on different imaging strategies. One imaging mode included numerous cameras mounted on fixed viewpoints to capture multi-view images around a plant, in which both plant and cameras were stationary [26]. This kind of installation has the highest imaging efficiency and is useful for high-speed imaging under field conditions. Other installations used the imaging mode of the camera in conjunction with a rotating table [27]. This kind of installation reduces the cost and the number of cameras by employing a turntable. A few cameras (usually two or three) might result in limited imaging perspectives, which might not apply to specific plants with other structural characteristics. A fully automated plant phenotyping cell comprising a six-axis robot and a high-precision turntable has been used as an active vision approach to intelligently capture images [28]. This imaging mode can flexibly acquire images needed for the reconstruction of target plants, tuning camera placement to match specific plant structures. This method is more accurate and requires fewer images than static imaging approaches. However, with the size restrictions related to the reach of the robotic arm, it might not be suitable for large plants. The complexity of plant structure may vary widely from the vegetative to the reproductive stage of the life cycle. To further facilitate the faithful reconstruction of large and/or complex plants, the proposed imaging system increased the number of imaging perspectives to obtain more detailed 3D information. Therefore, the imaging system achieved a balance between the cost of mechanical device/imaging sensors and the number of imaging perspectives that could be extended to the high-quality 3D reconstruction of larger and/or more complex plants (e.g., mature densely grown roots or shoots) (Fig. 4 and Additional file 1: Fig. S4).

Compared with the popular vision-based 3D root phenotyping systems for plant seedlings grown in transparent media under controlled environments (e.g., the gel system [7]), our study transformed plant cultivation from non-soil to soil medium and extended the observation period to the mature stage. In addition, we used more cameras as well as more imaging perspectives to reconstruct the complex 3D RSA at maturity with high resolution. Compared with the 3D root (soil-grown) measurement systems in the lab (e.g., X-ray and MRI), the advantages of our system are: (1) improved efficiency; (2) low to no disturbance of complex soil environment to 3D root reconstruction; (3) reduced investment in hardware and software; and (4) extended observation period of root size to late growth stage. Compared with the vision-based 3D root phenotyping system for mature field-grown roots (DIRT3D) [21], our method has expanded the observation from only the root crowns of the root base at the mature stage to the whole root system from seedling to the mature stage. In DIRT3D, the root systems were held horizontally during imaging. The mechanical support strength of the root crowns of mature field-grown maize is strong enough to maintain its structure in the absence of soil support. However, for most crops, the root diameter at the seedling is smaller and the supporting strength is weaker, and its structure will be altered after losing soil support, especially for the lateral root of the tap root system. We adopted a mesh system [29] upon which plants can be grown and the RSA can be maintained during imaging. The stability and rigidity of the mesh system were improved by using stainless steel; thus, it was strong enough to support the large root systems of mature plants. Moreover, we specifically designed and adopted the combination of the fan-shaped and vertical arrangement of cameras, as it provided a more appropriate imaging perspective for the root of the support mesh system, to facilitate the later 3D reconstruction processes. The plant rotating mode saves imaging space compared to the camera rotating mode. In addition, our method was suitable for obtaining phenotypic information for both aboveground and underground plant parts (Additional file 1: Fig. S4).

The strengths of our method lie in its cost-effective, efficiency and convenience, and its adaptability to study different plant species (monocotyledons and dicotyledons), different plant parts (root and shoot), different growth stages, and different experimental conditions, such as drought stress or effects of soil resources on root angle, depth, and distribution. For RSA analysis to be effective at the genome scale and contribute to plant improvement efforts, the imaging platform must have the capacity to phenotype thousands of plants. The time for measuring thousands of plants with only one imaging system would very likely limit the throughput and efficiency considerably. For systematic screening of a large number of genotypes, it needs dozens of such imaging systems. Our imaging system costs about 4600 US Dollars in total for a standard device with twelve camera sensors. From the perspective of economic and cost/benefits, the imaging cameras we used are cheap and have stable operation and fast imaging and transmission capabilities, which enables the continuous imaging of thousands of plants. As the servomotor and the main imaging control module were controlled by the commercial PLC, the system can run stably in high-intensity continuous operation and can be integrated into a middle-size PLC for batch control of dozens of imaging systems. In addition, the imaging system is very easy to use and is a high efficiency (3 min per plant), which greatly reduces the costs of time and labor for high-throughput 3D root imaging. Moreover, it can be easily customized or replicated to meet the needs of various users. These features help to increase the scalability of our system, as more devices can be built relatively cheaply and flexibly to accommodate more experiments and advance high-throughput root phenotyping.

The proposed system also has a few disadvantages. The fine roots might break and the root trajectory might slightly change despite being supported by the support mesh. Due to destructive sampling, the continuous observation of the root system of the same plant is not possible. The 40 cm diameter of the PVC pipes might constrain and alter the RSA of the tap root system. A larger container was required to obtain a more precise growth trajectory of roots.

The 3D reconstruction method used in this study was effective in obtaining a digital description of 3D RSA. The redundant background information on each image was eliminated after using Masquerade (a tool included in 3DF Zephyr used to mask out the background pixels automatically, detailed information in Methods). Thus, the false feature matching and the amount of data transferred to online storage were effectively reduced, and the efficiency of 3D reconstruction was finally improved.

### Quantification of root distribution and root traits enhances comprehensive 3D RSA analysis

Various root architecture parameters, including global and local root traits, could be automatically extracted using our pipeline (Figs. 5, 9, and 10). Therefore, the 3D root architecture could be quantitatively described, providing a way for further research on nutrient absorption by plant roots. The global root traits extracted in this study, including root depth, convex hull volume, surface area, volume, and total length, had a good correlation with root dry weight (Fig. 6A and C–F). It implied that the global root architecture parameters, such as basic phenotypic traits, could be used not only as an intuitive indicator of root size and root growth state but also for the estimation of root biomass, which is important for biological research and agricultural management.

According to the correlation of the extracted and measured values of the total root length (Fig. 6I), it can be inferred that the total root length was underestimated for the late growth/mature stage, which might be due to that some roots were merged into one after voxelization. However, it was more likely due to that the roots of the high branching zone were shaded from each other. In addition, the disconnected roots caused by the removal of the support mesh were also one of the reasons for the underestimation of the total length. The total volume and surface area of the root systems were likely to be underestimated due to similar reasons. According to the comparison of the extracted and measured values of the length and diameter of the main root and lateral roots of rapeseed and the nodal roots and stem of maize (Fig. 11A–D), the estimation error was in an acceptable range (rRMSE < 7%).

The reconstructed 3D root model enabled us to retrieve detailed data about the distribution of the root system as a function of soil depth and horizontal distance from the root base. The visualization of the spatial distribution of RLD provides a fast and semi-quantitative method of assessing how individual plants are distributing roots in soil (Fig. 7A). The vertical and horizontal RLD distribution of individual plants enables the statistical comparisons of root distribution across genotypes. The vertical and horizontal distribution of roots of different genotypes showed no significant difference at the early growth stage (stages 1–2), but a significant difference at the late growth stage (stages 4–5) (Fig. 7B, C). At stage 4, for example, NY22 was much denser in the top 10 cm soil layer while NZ1818 was denser in the 10–30 cm soil layer. The root length density was calculated based only on visible roots, the values might be underestimated due to the mutual occlusion of roots, especially for the dense branching zone.

The extraction of local root traits (e.g., length, diameter, and angle of lateral/nodal roots) requires segmenting the reconstructed 3D root model into individual roots. In this study, we introduced an automated method to reliably segment different root types (Figs. 8 and 9A, E). Automated root segmentation was difficult for dense roots because of occlusion among lateral or nodal roots. Automated root segmentation has been achieved in 2D root phenotyping and most of them are based on topological analysis, such as RootReader2D [30], RootSystemAnalyser [31], and RhizoChamber-Monitor [32]. However, fully automated segmentation of different root types in 3D remains a challenge, particularly for mature soil-grown crops with extremely complex topologies. In recent years, several powerful 3D root traits extraction tools have been developed, such as DynamicRoots [33], DIRT3D [21], TopoRoot [34], and 4DRoot [35]. DynamicRoots can produce a full branching hierarchy and associated root traits, while it is designed for a time-series of simple seedling-stage roots. Its accuracy can be significantly affected by the disconnected and loop root components which are abundant in 3D images of mature roots. TopoRoot can obtain the complete hierarchy and root traits of a mature maize root system from a single 3D image. It can deal with topological errors without the need for a time series. This software identifies the stem (hierarchy 0) of maize based on the thickness of the skeletons, and makes an important assumption that the roots higher up in the hierarchy are generally longer. However, for tap root systems (take rapeseed for example), there was no significant difference between the diameter of some parts (middle to apical) of the main root and that of lateral roots (especially near the base of laterals) (Figs. 8A and 9A), and many first-order lateral roots are longer than the main root (Figs. 8A and 9A). Moreover, some roots were disconnected due to the removal of the support mesh, which would affect the hierarchical analysis. Therefore, TopoRoot may not be suitable to identify the main root and lateral roots for our 3D root model of rapeseed. Nevertheless, it is still outstanding in obtaining the complete root hierarchy and fine-grained traits of mature maize roots. 4DRoot can extract root traits and hierarchy based on 3D surface geometry. However, 4DRoot was not powerful at the segmentation of lateral/nodal roots near the root base compared with our developed algorithm (Additional file 1: Fig. S5, indicated in a red circle). But it is still a very powerful and easy-to-use 3D root architectural analysis software. DIRT3D enables the accurate extraction of 3D root traits based on the 3D point cloud at the individual and crown levels. It identifies the individual roots on each slice using active contour snake method, and then connects them to obtain the root trajectories. DIRT3D was also used to process the same point clouds of our root systems of maize and rapeseed (Additional file 1: Fig. S5), and the result showed that the identification and skeleton extraction of lateral/nodal roots near the root base was not precise. However, this software can reconnect roots that were disconnected by removing the support mesh. In this study, we combined the methods of slice segmentation and skeletonization to extract the 3D root traits for tap root systems and fibrous root systems. A horizontal slicing and iterative erosion and dilation method was developed (Fig. 8) and was effective in the automated segmentation of different root types (Figs. 8 and 9A, E). For the upper slices, the main root was coarse and the lateral roots were fine. Through appropriate erosion and dilation, the lateral roots were eliminated and only the main root was retained. While, for the lower slices, there was little difference between the diameter of the main root and lateral roots. The lateral roots were eliminated by determining the initial location of the main root based on the main root of the upper slice. The developed method enables automated root segmentation, and then the skeleton of the lateral roots/nodal roots could be extracted directly to calculate the local root traits, including the initial angle of lateral/nodal roots. The developed algorithm also has some limitations. It was more suitable to segment the root systems where lateral roots were obviously thinner than the main root. However, for root systems with very close main root and lateral roots diameters, or with obvious and rapid bending main root, it may fail to obtain a satisfactory segmentation result.

The initial angles combined with the gravitropic responses of individual roots can affect the spatial distribution of the root system and can ultimately influence the capability of a plant to access and acquire water and nutrient resources [2]. The root angle calculated from the root trajectory of the real root system is a valuable phenotype for parameterization of the 3D root model and for distinguishing genotypic variation. The number of nodal roots can also be a key factor for water and nutrient uptake [33, 36]. However, these root traits cannot be accurately measured by traditional methods. Most root angles and root numbers estimated in 2D images are susceptible to measurement errors [37]. The growth trajectory of the roots was altered by the limited growing space, and the dense nodal roots overlapped and obscured each other. The information encoded in the shape of root systems (e.g., root angles and the number of nodal roots) could be obtained by the method described in this study (Figs. 9D, H and 10A, D, F). The proposed 3D root phenotyping pipeline provides a tool to understand the various changes in root architecture, which facilitates breeding for favorable root characteristics to improve yield in suboptimal conditions, including drought and low soil fertility.

## Conclusion

The automated imaging system proposed in this study efficiently acquired multi-view images (3 min per plant) and successfully reconstructed the 3D architecture of the maize and rapeseed root systems. The global and local root traits (e.g., length, diameter, initial angle, and the number of lateral/nodal roots) were obtained automatically for investigating 3D RSA. Detailed analysis of the Spatio-temporal distribution of RLD enabled the comparisons of root distribution across genotypes. The strengths of our method lie in its low cost, high efficiency, and low labor intensity; relatively high root data integrity (complete root can be observed and measured); including 3D spatial data (e.g., root distribution and root gravitropism/initial angle) and temporal data (from vegetative to reproductive stage); and suitable for both monocotyledonous (e.g., maize) and dicotyledonous (e.g., rapeseed). The enhanced 3D visualization and quantification capabilities of our 3D root phenotyping pipeline can enable comprehensive 3D RSA analysis and propel advances in crop productivity.

## Methods

### Root growth system

A stainless steel root support mesh was constructed to support the root system and retain the 3D RSA (Fig. 2A). The root support mesh was composed of mesh discs with a diameter of 0.24–0.38 m and a grid of 10 mm × 10 mm (Fig. 2B), threaded rods with a height of 0.4–1.0 m, and flange nuts. Mesh discs were located 0.10–0.15 m apart, and each mesh disc was anchored to 3–4 threaded rods using 6–8 stainless steel flange nuts (Fig. 2A). The top mesh disc included an opening in the center to accommodate the girth of the taproot, which is the most at the plant base. The constructed root support mesh was painted black using a black waterproof matte paint.

A demountable polyvinyl chloride (PVC) pipe with a diameter of 0.3–0.4 m and a height of 0.4–1.0 m was used for plant cultivation. The PVC pipe was composed of two detachable half-cylinders (Fig. 2C), which were separated by unloading four pipe hoops. The PVC pipe was placed in the field, and the root support mesh was inserted into the PVC pipe. Subsequently, the PVC pipe was filled with a 2-mm sifted soil mixture containing 50% local paddy soil and 50% river sand. Seeds were planted at a depth of 30–40 mm in the center of the PVC pipe.

### Automated imaging system

An automated imaging system was designed and constructed to rapidly capture multi-view image sequences (Fig. 1). The customized automated imaging system included a rotatable imaging arm, a pedestal, and a black background plate (Fig. 1A). The customized imaging arm was composed of an arc arm, with a radius of 1.5 m and an angle of 90°, and a vertical arm with a length of 0.5 m. Twelve low-cost, highly versatile imaging cameras (HIKVISION DS-2CD3T86FWDV2-I8S 4 K Outdoor IR Fixed Network Bullet Camera) were mounted at 10° intervals on the arc arm and 0.10–0.15 m intervals on the vertical arm. The imaging arm was mounted on one end of the pedestal, while a black background plate, with a width of 0.8 m and a height of 1.5 m, was mounted on the opposite side of the pedestal.

In the preliminary experiments, other camera arrangement strategies (Additional file 1: Fig. S5) were tested, including the vertical arrangement of the cameras parallel to the plant root, and the approximate fan-shaped arrangement of the cameras focusing on the plant root. The results showed that the combination of the fan-shaped and vertical arrangement of cameras with circular movement trajectory outperformed the other two strategies for the 3D reconstruction of plant root systems (data not shown).

Two imaging movement modes were designed to capture multi-view images, one was that the camera stayed stationary while the plant rotated, and the other was that the cameras rotated around the static plant. To rotate the plant or the imaging arm, the rotary table and the pedestal were respectively fixed on the top of two separate slewing bearings that were driven by a high-resolution alternating current servomotor (750 W, 3000 rpm; SDGA-08C11BB; Tode Technologies Co., Ltd.) and gear with 0.005° repositioning resolution (Fig. 1A). The movement of the servomotor, which was plugged by gear reduction with a reduction ratio 1:10, was controlled by a programmable logic controller (PLC).

An imaging control box (ICB) was mounted on the opposite side of the imaging arm (Fig. 1B and C). It was mainly used for imaging control, image acquisition, real-time image display, online debugging, and data recording and storage. A ground base station (GBS) was configured to implement movement control, parameter settings, real-time image display, and image acquisition and downloading (Fig. 1B and D).

The movement of the automated imaging system was controlled by the PLC installed on the GBS. A 10-inch human–machine interface (HMI) mounted on the GBS was used to control the automated imaging system and configure the parameters of rotation (e.g., the rotation rate and rotation speed of the motor, direction of movement, and the angle of rotation per image set capture) (Fig. 1D and Additional file 1: Fig. S6).

The automated imaging control system was composed of the PLC, a camera imaging controller (32-channel relay serial port module), and 433 MHz wireless data transmission (Fig. 1B, C, and Additional file 1: Fig. S6). The PLC of the GBS relied on 433 MHz wireless data transmission for communication with the ICB, which was used to control the switch of the camera imaging controller (relay) of the ICB. The ICB relay was connected to the alarm input interface of the camera to trigger the shutter of the camera. A power over Ethernet (POE) network switch (24 channel full gigabit) and a solid-state disk video recorder (DVR, 16 channel network DVR with 4TB hard disk drive) were installed on the ICB. All the cameras were connected to the POE switch. The POE gigabit switch was connected to the solid-state DVR to realize image transmission and storage.

A visual display and touch panel installed on the ICB were connected to the solid-state DVR. This ICB display could simultaneously display real-time images and videos from up to 16 cameras. The ICB touch panel was used to control the display screen to select an individual camera image for a larger window view, which was used to check the focus and clarity of the camera lens. A 5-inch HMI mounted on the ICB was connected to the camera imaging controller. Each camera could be switched on or off using the ICB HMI. With the HDMI wireless video transmission between the ICB and the GBS, real-time images were displayed on the GBS display. The POE gigabit switch of the ICB was connected to the AP gigabit network bridge. The industrial control computer in the GBS remotely accessed the solid-state DVR of the ICB and acquired images by the AP gigabit network bridge.

Before automated imaging, manual focus was used to adjust the camera lens for shooting clear and sharp images. All the cameras were set with 6-mm focal length and 3840 × 2160 pixels. The camera configurations, such as aperture (F4.0) and shutter speed (1/10 s), were set using iVMS-4200 software (HIKVISION). The camera imaging controller synchronized the images captured. The images of all cameras were automatically transferred to the solid-state DVR storage. The server unit stored information about the static IP user account. It used the ONVIF protocol to transfer the images from each client unit to the DVR storage. Images were downloaded and transferred to computers through the same network using the iVMS-4200 software (https://www.hikvision.com/en/products/software/ivms-4200/?q=ivms4200%20series&position=1).

### 3D reconstruction and data preprocessing

Multi-view image sequences captured by the imaging system were imported into the 3DF Zephyr Aerial proprietary software (v.4.530; 3DFlow, Verona, Italy) [24, 38] for the 3D reconstruction of individual root systems. This software uses SFM and MVS photogrammetry to recover 3D structures from image sequences [24, 38]. Briefly, features were identified and matched across multiple images to estimate the parameters of mathematical camera models and camera positions and orientations based on epipolar geometry. These estimates were then used to generate a sparse point cloud of the tie points with 3D coordinates, which were optimized by a self-calibrating bundle adjustment. The self-calibrating bundle adjustment was used to improve the initial estimates of structure (e.g., 3D positions of features) and camera pose parameters in SFM by minimizing a cost function of the difference between the projection of the feature points and the tracked features across images. Given the generated sparse point cloud with the established epipolar geometry, the pixel-wise disparity was computed to reconstruct a disparity map for each image in the image sequence. The pixels were then back-projected to all overlapping images and triangulated to generate a dense point cloud [23, 39].

To improve the efficiency and accuracy of the 3D reconstruction, a mask was applied to remove background pixels from root images. 3DF Masquerade (a fully automatic background pixels removal tool included in 3DF Zephyr, https://www.3dflow.net/zephyr-doc/en/3DFMasquerade.html) was used to mask out the background pixels. Simply pick the color of the background and all the colors that fall behind a certain (customizable) threshold can be selected and deleted automatically. This tool omitted the redundant background information, decreasing false feature matches during 3D reconstruction.

The 3DF Zephyr toolbox was used to orient the point clouds by manually defining the up vector assuming the plant growing from the bottom down (the negative z-axis). Two features/points on the support mesh were manually selected to measure the distance of the mesh diameter, and then convert the 3D point clouds to the actual size according to the actual size of the support mesh. Using the selection tool of 3DF Zephyr (https://www.3dflow.net/technology/documents/3df-zephyr-tutorials/tutorial-cleaning-point-clouds/), the 3D point cloud of the black root support mesh was removed by selecting all the points of a given color (within a given threshold). Some of the reflection points of the support mesh need to be manually selected and removed, which took about 3–10 min according to different cases (the size of the support mesh and the number of reflection points). In addition, noise and outliers were removed by a statistical filter tool of 3DF Zephyr, which can select and remove all points below a certain confidence threshold to obtain a smoother dense point cloud.

### Extraction of root traits

A 3D point cloud processing pipeline was developed to automated extract the root traits. 3D root traits could be extracted in four steps: (1) *Voxelization* A 3D root voxel model was generated from the 3D root point clouds. In this step, different voxelized cell sizes should be pre-set, and the most appropriate cell size and voxelized model should be selected by visually contrasting the 3D point clouds and the 3D root model after voxelization; (2) *Global root traits extraction* Global root traits were extracted from the point clouds and voxels automatically. Root Depth, Width and Convex Hull Volume were extracted based on the point clouds. Root volume, surface area, and root skeleton were extracted based on the root voxels. The total root length was computed from the extracted root skeleton; (3) *Root segmentation* The voxels of different root types were segmented from the whole 3D root voxels. A key parameter, the percentage of the amplified area based on the main root on the upper slice, should be set in this step. This parameter was selected empirically based on the curvature of the main root; (4) *Detail root traits extraction* Detailed root traits were extracted from the voxels of different root types. Root skeletons of different root types were extracted based on the voxels. Then, the length, diameter, angles, and so on can be computed from the skeleton of different root types. The processing time was about 4–7 min, including steps 1 (about 3–5 min) and steps 2-4 (about 1–2 min).

The 3D point clouds of root systems were converted to 3D root voxels by a transformation algorithm/function *pnt2vox* in PAREIDOLIA [40]. The voxelization of the 3D root point cloud was sensitive to cell size. If the selected cell size was too small, it could not form a continuous single root (Additional file 1: Fig. S8D, (3)) or could form some holes on thick roots (Additional file 1: Fig. S8D, (4)); if the selected cell size was too large, the roots would become thicker (Additional file 1: Fig. S8B, (1)) and the adjacent roots would be merged into one (Additional file 1: Fig. S8B, (2)). The proper cell size used in voxelization was empirically selected by visually contrasting the voxelized root systems with the 3D root point clouds (Additional file 1: Fig. S8). The selection criterion was: (a) no obvious change in root diameter (Additional file 1: Fig. S8B and C, (1)); (b) most individual roots could form a continuous root without holes (Additional file 1: Fig. S8C and D, (3) and (4)); and (c) there were fewest roots be merged into one (Additional file 1: Fig. S8B and C, (2)).

The maximum vertical depth and horizontal width of the point cloud of the whole root system were regarded as the root depth and root width, respectively (Fig. 5A). A quickhull algorithm [41] was used to build a convex hull and calculate the convex hull volume for the whole root system (Fig. 5B). The root volume was calculated based on the count of the actual number of “on” voxels in the 3D root voxels, and the root surface area was calculated based on the distance around the boundary of the 3D root voxels (Fig. 5C). The 3D skeleton of root systems was extracted using the optimized homotopic thinning algorithm [42], and the total root length was calculated based on the skeleton (Fig. 5D).

The different types of roots were automatically segmented by using the developed horizontal slicing and iterative erosion and dilation algorithm (Fig. 8). The skeleton of the main root/stem and lateral/nodal roots was also extracted using the optimized homotopic thinning algorithm, and the total length was calculated based on the skeleton (Fig. 9B, F). The average diameter of the main root/stem and lateral/nodal roots was calculated by the formula $SA/\pi \cdot TRL$ or $2\cdot\sqrt{V/(\pi \cdot TRL)}$ (*V*: volume; *SA*: surface area; *TRL*: total root length). The initial angle of lateral/nodal roots was extracted by calculating the vertical angle of the vector from the point 10–15 voxels away from the start point.

### Plant materials and growth conditions

The experiment was performed at the farm of Jiangsu Academy of Agricultural Sciences, Nanjing, China (32.03° N, 118.87° E) in 2020–2021. Two rapeseed (*Brassica napus* L.) cultivars “Ningyou 22” (NY22, conventional) and “Ningza 1818” (NZ1818, hybrid), and one maize (*Zea mays* L.) cultivar “Sukeyu 1705” were used. The field management, such as fertilization, weeding, and pest control, followed the local cultivation practices. Two rapeseed cultivars at five growth stages (Additional file 2: Table S1) and one maize cultivar at the filling and mature stages were selected for root imaging and reconstruction. Three representative plants were used per species, per growth stage.

We imaged the maize root system as a whole, as well as those with the lateral roots of nodal roots removed, respectively. The global root traits were extracted using the root systems imaged as a whole, while the local root traits were extracted using the imaged root systems with the lateral roots removed. For root trait validation, we used a scale to measure the depth of root systems. All roots of the same plants above were destructively sampled and scanned with a flatbed scanner (Epson Perfection V800, Japan) at a resolution of 600 DPI. A WinRHIZO Pro 2013 (Régent Instruments, Canada) image analysis system was used to analyze the scanned root images. The length and diameter of roots were calculated according to the method of Wu et al. [43]. The root angle was measured manually using a protractor. The measured angle was the vertical angle formed from the root base to a distance of 3–5 cm away from the root base. Usually, we start with easy-to-measure roots and then cut them off to reduce interference with other root angle measurements. The measured roots were marked according to their position on the stem or main root. All roots were oven-dried for 30 min at 105 °C, then at 80 °C until reaching a stable weight. Dry weight was measured using a 0.001-g electron balance.

## Supplementary Information

**Additional file 1: Figure S1**. Extraction of global root traits, including root depth, width, width/depth, convex hull volume, surface area, volume, total length, and solidity of mature maize at two sampling stages. **Figure S2.** Spatial distributions of root density for adult maize (A) and adult rapeseed (B). **Figure S3.** Examples of the failure of 3D reconstruction using manually acquired images. **Figure S4.** Reconstructed 3D point clouds of four crops at different growth stages. Reconstructed shoots of rapeseed seedling (A), flowering rapeseed (B), rice seedling (C), mature rice (D), mature wheat (E), and cotton (F). **Figure S5.** The segmentation of the different types of roots of rapeseed (A) and maize (B) by using our developed algorithm, 4DRoot and DIRT3D, respectively. **Figure S6.** The arrangement of the camera position of three different image acquisition strategies. A. Vertically arranged the camera position parallel to the plant root; B. Approximate fan-shaped arranged of the camera position centering on the plant root; C. Fan-shaped arranged of the camera position centering on the upper part of the root and vertically arranged of the other camera position parallel to the root support mesh. **Figure S7.** Design of the multi-camera automated imaging system. **Figure S8.** The 3D point clouds (A) and the voxelized 3D model by using different cell size (B-D) of rapeseed root system.

**Additional file 2: Table S1.** Information on the five growth stages.

## Data Availability

The datasets supporting the conclusions of this article, and 3D root traits extraction pipeline, test data, as well as a brief user guide, are available at https://github.com/wuqiangithub/AutomatedRotatingImagingSystem.

## Abbreviations

RSA: Root system architecture
MRI: Magnetic resonance imaging
SFM: Structure from motion
MVS: Multi-view stereo
CHV: Convex hull volume
V: Volume
SA: Surface area
TRL: Total root length
PVC: Polyvinyl chloride
ICB: Imaging control box
GBS: Ground base station
HMI: Human–machine interface
PLC: Programmable logic controller
POE: Power over Ethernet
DVR: Disk video recorder
