# Supplementary material for: Quantification of the three-dimensional root system architecture using an automated rotating imaging system
Source: Plant Methods. 2023 Feb 2;19:11. doi: 10.1186/s13007-023-00988-1 (PMC9896698; doi:10.1186/s13007-023-00988-1)
Supplement: Supplementary file 1 — Additional file 1: Figure S1. Extraction of global root traits, including root depth, width, width/depth, convex hull volume, surface area, volume, total length, and solidity of mature maize at two sampling stages. Figure S2. Spatial distributions of root density for adult maize (A) and adult rapeseed (B). Figure S3. Examples of the failure of 3D reconstruction using manually acquired images. Figure S4. Reconstructed 3D point clouds of four crops at different growth stages. Reconstructed shoots of rapeseed seedling (A), flowering rapeseed (B), rice seedling (C), mature rice (D), mature wheat (E), and cotton (F). Figure S5. The segmentation of the different types of roots of rapeseed (A) and maize (B) by using our developed algorithm, 4DRoot and DIRT3D, respectively. Figure S6. The arrangement of the camera position of three different image acquisition strategies. A. Vertically arranged the camera position parallel to the plant root; B. Approximate fan-shaped arranged of the camera position centering on the plant root; C. Fan-shaped arranged of the camera position centering on the upper part of the root and vertically arranged of the other camera position parallel to the root support mesh. Figure S7. Design of the multi-camera automated imaging system. Figure S8. The 3D point clouds (A) and the voxelized 3D model by using different cell size (B-D) of rapeseed root system. [file 13007_2023_988_MOESM1_ESM.pdf]

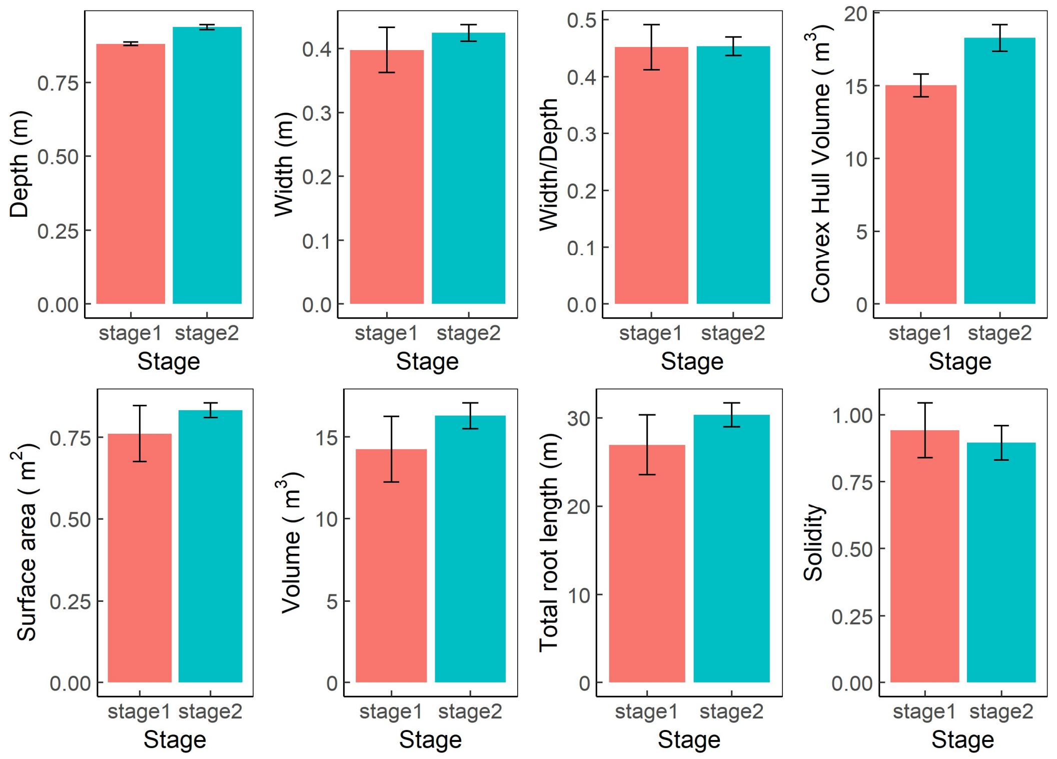

Figure S1. Extraction of global root traits, including root depth, width, width/depth, convex hull volume, surface area, volume, total length, and solidity of mature maize at two sampling stages.

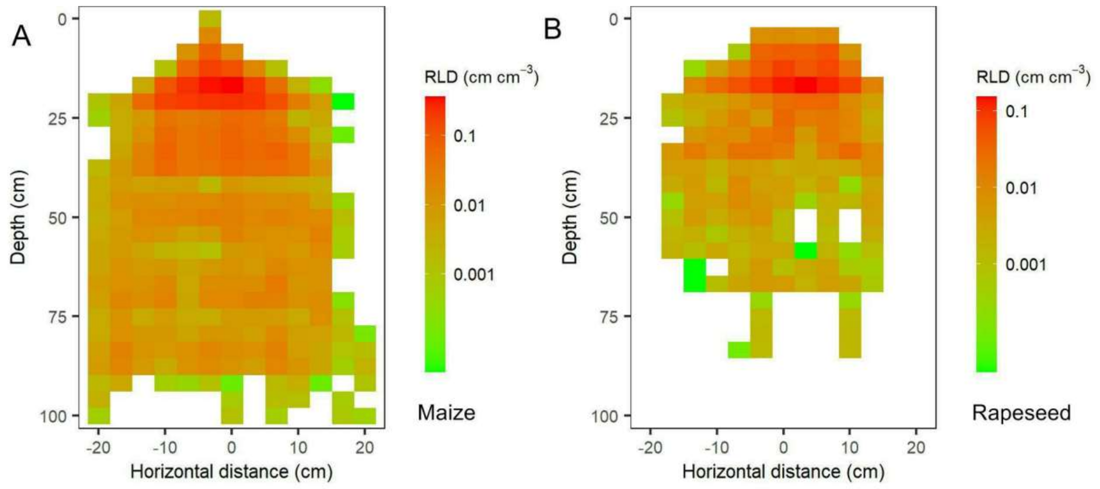

Figure S2. Spatial distributions of root density for adult maize (A) and adult rapeseed (B).

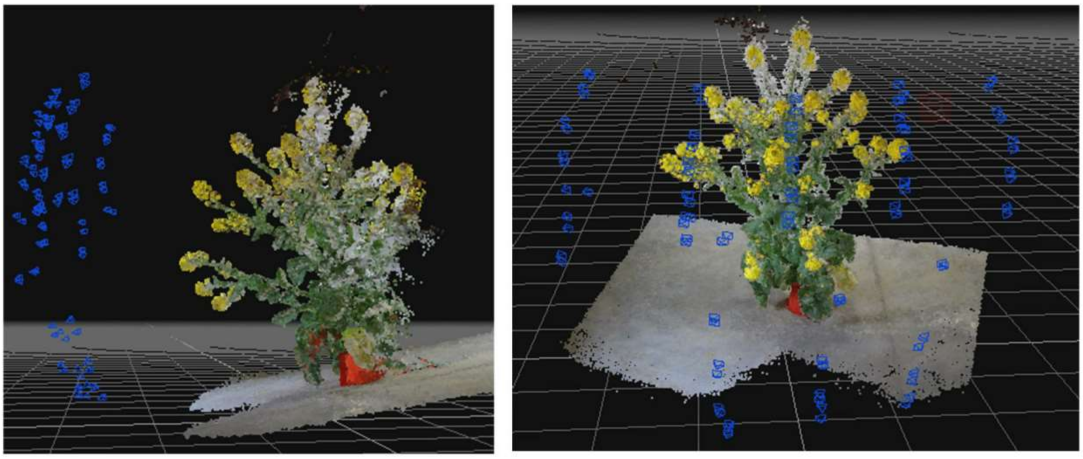

Figure S3. Examples of the failure of 3D reconstruction using manually acquired images.

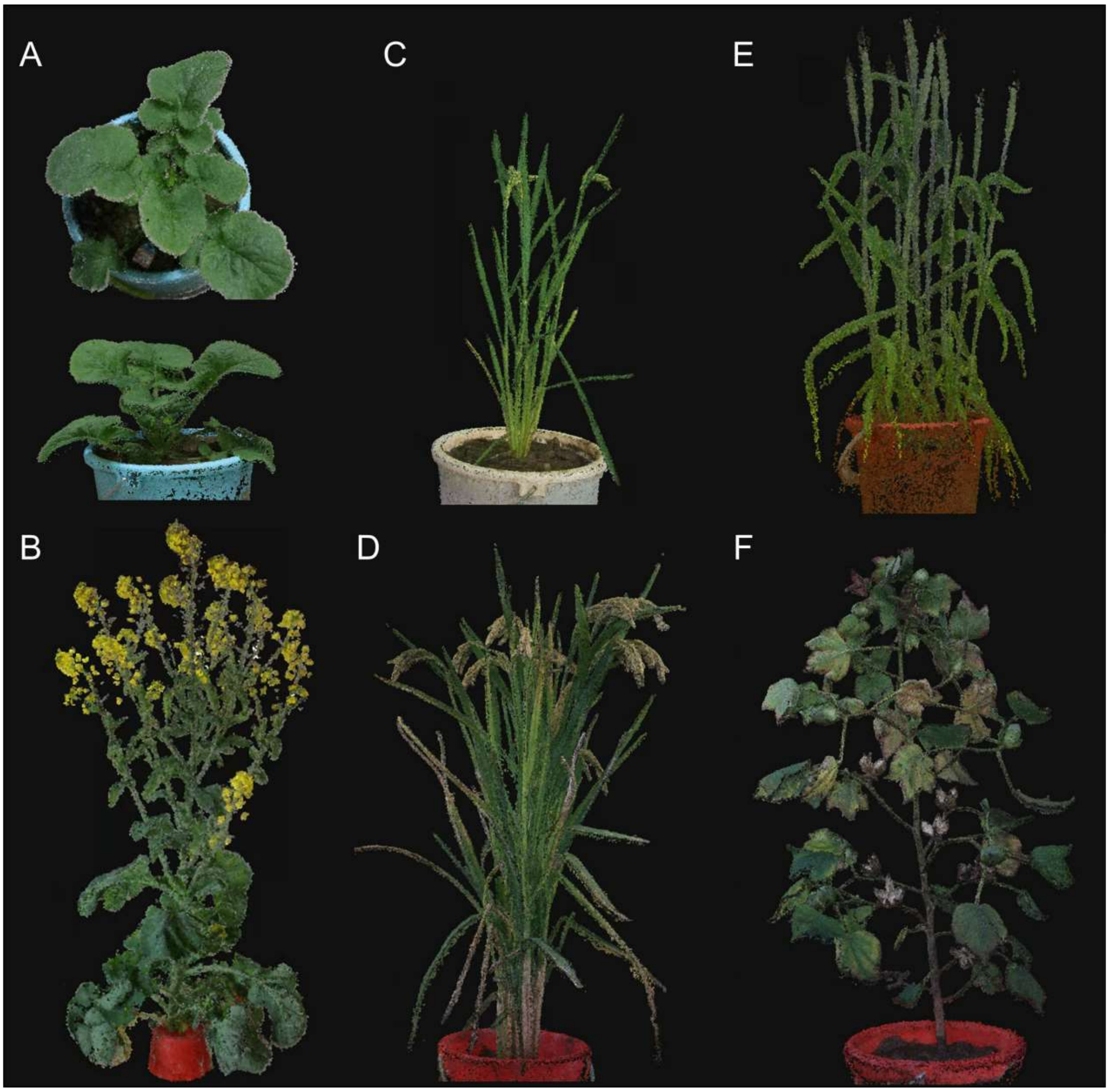

Figure S4. Reconstructed 3D point clouds of four crops at different growth stages. Reconstructed shoots of rapeseed seedling (A), flowering rapeseed (B), rice seedling

(C), mature rice (D), mature wheat (E), and cotton (F).

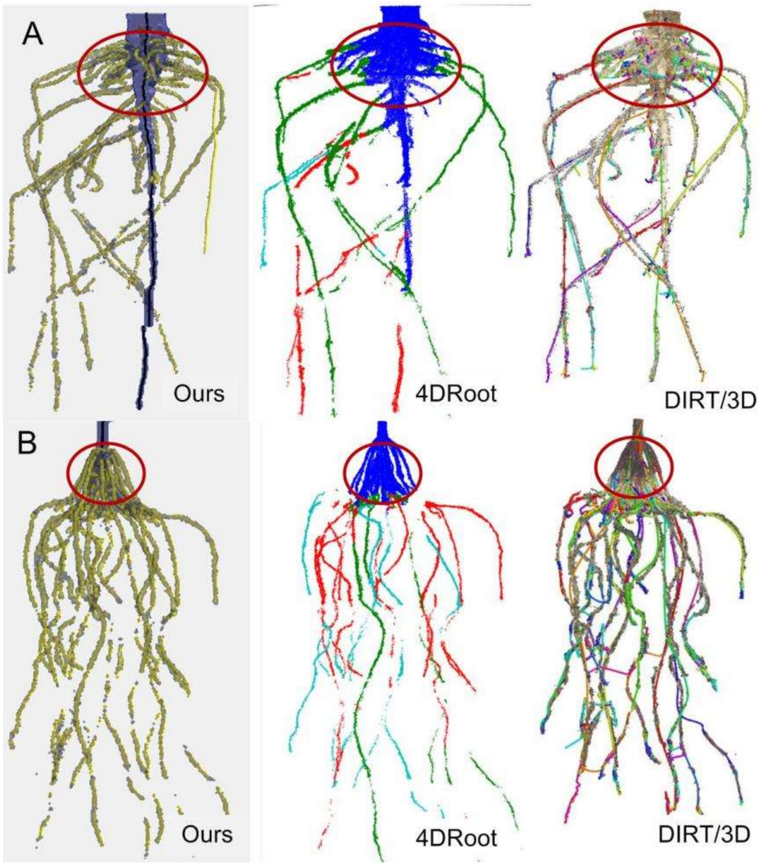

Figure S5. The segmentation of the different types of roots of rapeseed (A) and maize (B) by using our developed algorithm, 4DRoot, and DIRT/3D, respectively.

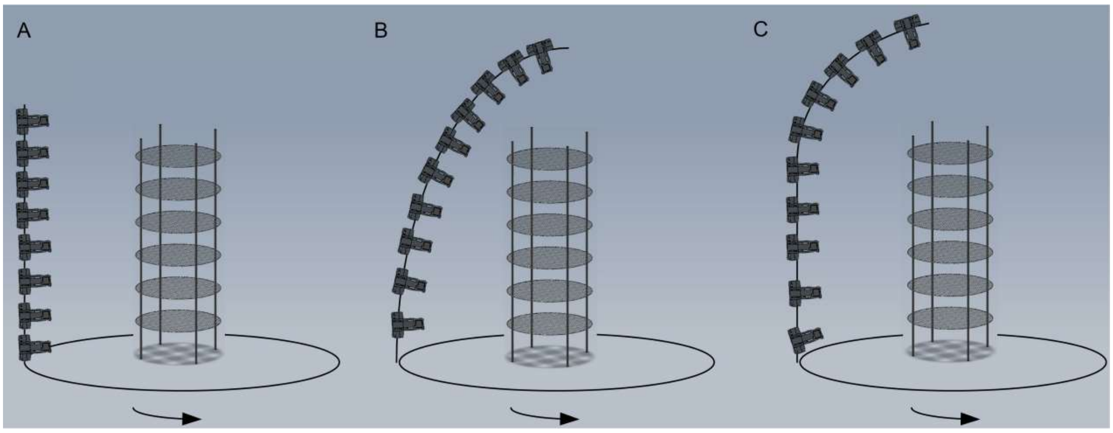

Figure S6. The arrangement of the camera position of three different image acquisition strategies. A. Vertically arranged the camera position parallel to the plant root; B. Approximate fan-shaped arranged of the camera position centering on the plant root; C. Fan-shaped arranged of the camera position centering on the upper part of the root and vertically arranged of the other camera position parallel to the root support mesh.

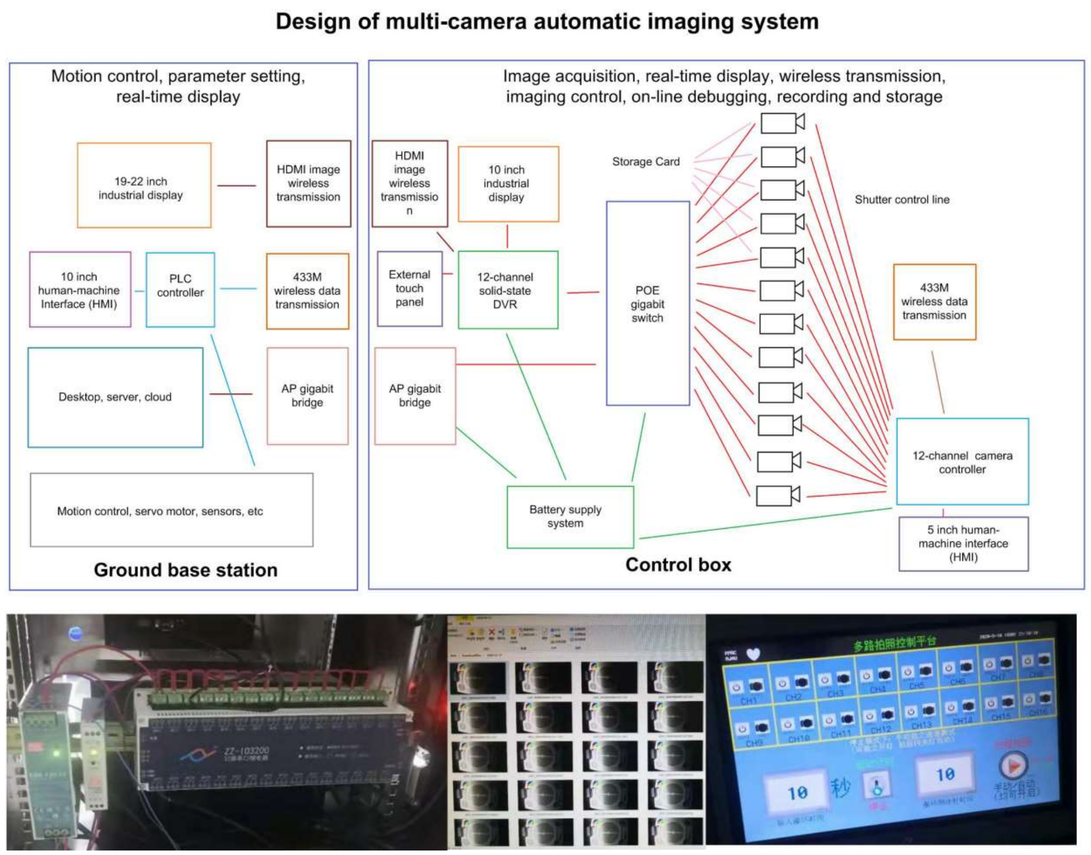

Figure S7. Design of the multi-camera automated imaging system.

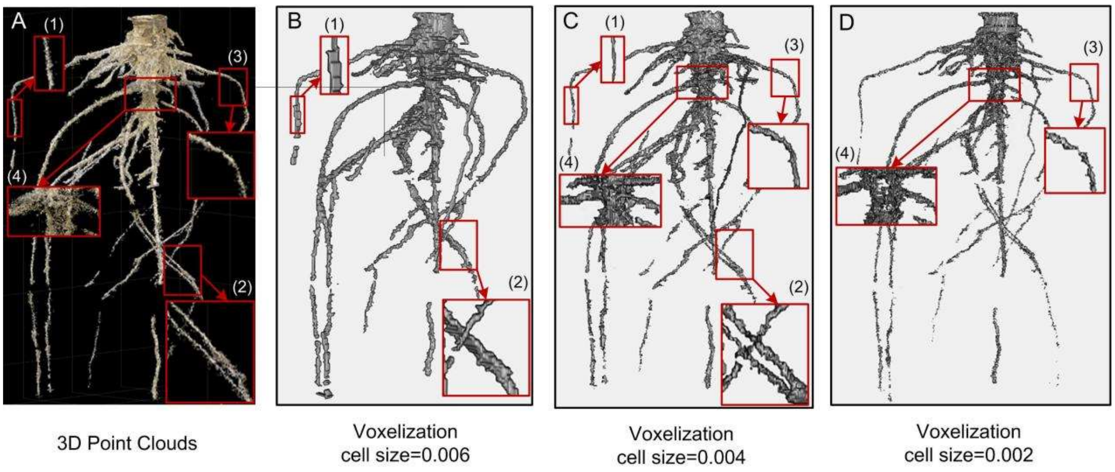

Figure S8. The 3D point clouds (A) and the voxelized 3D model by using different cell size (B-D) of rapeseed root system.
